# GSTM3 enhances radiosensitivity of nasopharyngeal carcinoma by promoting radiation-induced ferroptosis through USP14/FASN axis and GPX4

**DOI:** 10.1038/s41416-024-02574-1

**Published:** 2024-01-16

**Authors:** Yuting Chen, Yuanyuan Feng, Yanling Lin, Xiaohan Zhou, Lingzhi Wang, Yingtong Zhou, Kefan Lin, Longmei Cai

**Affiliations:** 1 Department of Radiation Oncology, Nanfang Hospital, Southern Medical University, 510515 Guangzhou, China; 2 Department of General Surgery, Nanfang Hospital, Southern Medical University, 510515 Guangzhou, China; 3 First Clinical Medical College, Southern Medical University, 510515 Guangzhou, China

**Keywords:** Radiotherapy, Head and neck cancer

## Abstract

**Background:**

Radiotherapy is a critical treatment modality for nasopharyngeal carcinoma (NPC). However, the mechanisms underlying radiation resistance and tumour recurrence in NPC remain incompletely understood.

**Methods:**

Oxidised lipids were assessed through targeted metabolomics. Ferroptosis levels were evaluated using cell viability, clonogenic survival, lipid peroxidation, and transmission electron microscopy. We investigated the biological functions of glutathione *S*-transferase mu 3 (GSTM3) in cell lines and xenograft tumours. Co-immunoprecipitation, mass spectrometry, and immunofluorescence were conducted to explore the molecular mechanisms involving GSTM3. Immunohistochemistry was performed to investigate the clinical characteristics of GSTM3.

**Results:**

Ionising radiation (IR) promoted lipid peroxidation and induced ferroptosis in NPC cells. GSTM3 was upregulated following IR exposure and correlated with IR-induced ferroptosis, enhancing NPC radiosensitivity in vitro and in vivo. Mechanistically, GSTM3 stabilised ubiquitin-specific peptidase 14 (USP14), thereby inhibiting the ubiquitination and subsequent degradation of fatty acid synthase (FASN). Additionally, GSTM3 interacted with glutathione peroxidase 4 (GPX4) and suppressed GPX4 expression. Combining IR treatment with ferroptosis inducers synergistically improved NPC radiosensitivity and suppressed tumour growth. Notably, a decrease in GSTM3 abundance predicted tumour relapse and poor prognosis.

**Conclusions:**

Our findings elucidate the pivotal role of GSTM3 in IR-induced ferroptosis, offering strategies for the treatment of radiation-resistant or recurrent NPC.

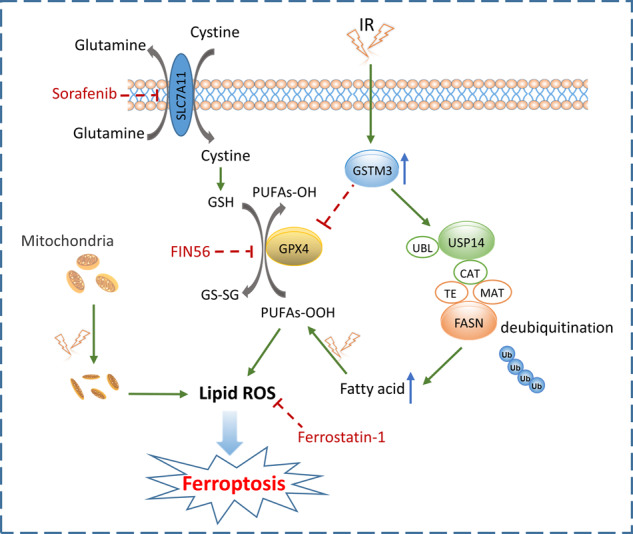

## Background

Nasopharyngeal carcinoma (NPC) is a malignancy originating from the nasopharyngeal epithelium and commonly diagnosed in southern China, Southeast Asia, and North Africa [1]. Radiotherapy plays a crucial role in the treatment of patients with non-metastatic NPC [2]. While the 5-year overall survival rate for NPC patients who have undergone standardised treatment has increased to 80–90%, approximately 10% of patients experience local recurrence due to radiotherapy resistance [3–5]. Radioresistance is attributed to diverse epigenetic regulatory mechanisms, including epithelial–mesenchymal transition, cancer stem cell properties, autophagy, and the oncogenic metabolism microenvironment [6–9]. However, the current understanding of these mechanisms does not fully address the challenges posed by radioresistance and tumour recurrence in NPC. Therefore, it is imperative to explore novel mechanisms that contribute to radioresistance and identify promising strategies to enhance the treatment response of NPC patients.

Ferroptosis is a distinct form of programmed cell death characterised by the involvement of intracellular iron and excessive lipid peroxidation, morphologically and mechanistically separating from other forms of cell death [10, 11]. Notably, ferroptosis plays a critical role in modulating radiotherapy sensitivity through diverse regulatory mechanisms in various cancers, including hepatocellular carcinoma, lung cancer, and melanoma [12–14]. Several classes of ferroptosis inducers (FINs) have been extensively studied and shown to effectively modulate cancer progression, regulate tumour microenvironment, and enhance treatment response [15–17]. Class I FINs, such as erastin and sorafenib, exert their effects by suppressing solute carrier family 7 member 11 (SLC7A11), limiting intracellular cysteine uptake and subsequent glutathione synthesis to induce ferroptosis. Class II and Class III FINs impede glutathione peroxidase 4 (GPX4) enzymatic activity and deplete GPX4 protein, respectively, hindering glutathione conversion and lipid hydroperoxide elimination. Targeting GPX4- or SLC7A11-induced ferroptosis regulates tumour development and enhances treatment response, offering a promising therapeutic approach for cancers [18, 19]. Itraconazole, cephalosporin, disulfiram/copper, and cucurbitacin B have been identified to possess potential antitumour activity in NPC by triggering ferroptosis [20–23]. However, the role of ferroptosis in sensitising NPC to radiotherapy has not been investigated in previous studies.

Glutathione *S*-transferase mu 3 (GSTM3), a member of the glutathione *S*-transferase family, exerts diverse effects on the progression of various malignancies [24]. On the one hand, GSTM3 influences the malignant metabolic pattern in pancreatic cancer, alleviates aggressiveness in renal cell carcinoma, and reverses radioresistance in hepatocellular carcinoma to suppress tumour malignancy [25–27]. On the other hand, specific genotypes of GSTM3 may confer increased susceptibility to cervical, colorectal, and prostate cancers [28–30]. Additionally, GSTM3 is associated with the malignant tumour behaviours and poor prognosis in colon cancer and glioma [31, 32]. However, the regulatory effects and detailed mechanisms of GSTM3 in NPC remain unclear.

In this study, we uncovered the crucial role of GSTM3 in facilitating ionising radiation (IR)-induced ferroptosis to enhance radiosensitivity in NPC. Mechanistically, GSTM3 acted by stabilising the ubiquitin-specific peptidase 14 (USP14)/fatty acid synthase (FASN) axis and directly inhibiting the expression of the glutathione peroxidase GPX4. The combination of FINs and IR treatment synergistically enhanced NPC radiosensitivity and inhibited tumour growth. Importantly, GSTM3 was correlated with radiotherapy response and predicted a favourable prognosis in NPC patients. These findings emphasise the significant role of GSTM3 in IR-induced ferroptosis and radiotherapy sensitivity, providing valuable insights for the development of promising treatment strategies targeting radiation-resistant or recurrent NPC.

## Methods

### Cell culture, cell transfection, and establishment of cell lines

Human NPC cell lines (5-8F, HONE1, and CNE2) were generously provided by Sun Yat-sen University Cancer Center, Guangzhou, China. The cells were cultured in RPMI-1640 medium (Gibco) supplemented with 10% fetal bovine serum (Gibco). All cell lines were maintained in a humidified chamber at 37 °C with 5% CO_2_.

For transfection experiments, small interfering RNA (siRNA) oligonucleotides (GemmaPharma) or plasmid DNAs (GeneChem) were transfected into the cells using Lipofectamine 3000 transfection reagent (Invitrogen) according to the manufacturer’s instructions. The specific sequences of the siRNA oligonucleotides are shown in Supplementary Table 1.

To establish a radiation-resistant cell line, CNE2 cells were repeatedly exposed to IR (6 Gy) until the fifth-generation surviving cells were obtained and designated as CNE2-R. To generate stable cell lines overexpressing GSTM3, cells were transfected with the Flag-GSTM3-GFP lentivirus synthesised by GeneChem. Puromycin (2 μg/mL) was used to select for cell colonies successfully transduced with the GSTM3-overexpressing lentivirus.

### Cell viability assays

NPC cells were treated with the designated concentrations of reagents or exposed to indicated doses of IR using a 6-MV X-ray beam. After 24 h, the culture medium was replaced with 100 μL fresh medium containing 10% Cell Counting Kit-8 reagent (FDbio Science), and cells were incubated for 1–4 h in an incubation chamber. The absorbance at 450 nm was measured using a microplate reader (Bio-Rad), and cell viability was calculated following the manufacturer’s instructions.

### Clonogenic survival assays

NPC cells were seeded in triplicate into six-well plates and exposed to varying doses of IR. The cells were incubated for 7–10 days until visible colonies formed. After washing with phosphate-buffered saline (PBS), the cell colonies were fixed with 4% paraformaldehyde for 15 min. Subsequently, the colonies were stained with 0.5% crystal violet (Macklin) for 30 min. Colonies with more than 50 cells were counted to calculate the surviving fraction. The curve of survival fraction (SF) with increasing dose (*D*) was plotted by the multi-target single-hit model: SF = 1– (1– *e*^[−*kD*]^) ^*N*^. The radiobiological parameters were derived: D0 = 1/*k*, Dq = D0 × ln (*N*), and SF2 = 1 – (1 – *e*^[−2*k*]^) ^*N*^.

### RNA extraction and quantitative reverse transcription PCR (qRT-PCR)

Total RNA was extracted from cell lysates using TRIzol reagent (TaKaRa Bio) and reverse-transcribed into cDNA using PrimeScript RT reagent Kit (TaKaRa Bio). qRT-PCR was performed using SYBR Premix ExTaqSYBR Green PCR kit (TaKaRa Bio). The Ct values were obtained and analysed using a LightCycler® 480 (Roche, Basel, Switzerland). β-Actin was used as a loading control. The primer sequences are listed in Supplementary Table 2.

### Lipid peroxidation assay

Cells were treated with FINs (5 μM FIN56 or sorafenib) or subjected to irradiation. After 24 h, the cells were washed twice with PBS and incubated with 500 μL of PBS containing 5 μM C11-BODIPY 581/591 dye (Invitrogen) for 30 min in the dark. The uncombined C11-BODIPY dye was removed by washing the cells with PBS. The fluorescence emitted by C11-BODIPY 581/591 was detected by simultaneously measuring the green (484/510 nm) and red (581/610 nm) signals using a flow cytometer (BD FACSAria III).

### Transmission electron microscopy (TEM)

The samples of cells were gently scraped using cell scrapers with 1 mL PBS and carefully transferred to 1.5 mL tubes. After centrifugation, the cell pellets were fixed with 500 μL of 2.5% glutaraldehyde at room temperature. After dehydration, embedding, and preparation of ultrathin sections, the samples were observed using a Hitachi H-7500 TE microscope.

### High-throughput targeted metabolomics of oxylipins

The cells in the test group were exposed to 6 Gy IR and then collected for high-throughput targeted metabolomics of oxylipins after 24 h. In brief, cell samples were processed using an extraction solution containing an isotopically labelled mixture for metabolite extraction. After homogenisation, sonication, and purification, the purified samples were evaporated to dryness and dissolved in 30% acetonitrile. The clear supernatant was subjected to ultrahigh-performance liquid chromatography-tandem mass spectrometry analysis. SCIEX Analyst Work Station (version 1.6.3) and Multiquant 3.03 software were employed for data acquisition and processing. High-throughput targeted metabolomics of oxylipins was conducted by Biotree Biological Technology (Shanghai, China).

### Whole transcriptome sequencing

After mRNA extraction, purification, and fragmentation, mRNAs were reverse-transcribed into single-stranded cDNA, and then the second strand of cDNA was synthesised. The cDNA was purified using Ampure Beads XP (Beckman). Subsequently, the ends of the cDNAs were repaired, and polyadenylation was added to the 3’-end. Sequencing adaptors were ligated to the cDNA ends, and PCR amplification was performed. After quality control, the library was subjected to paired-end sequencing using the Hiseq 2000 system (Illumina) with technical support provided by Shanghai Biotechnology Corporation.

### Protein extraction and western blot analysis

The pre-processed cells were washed twice with PBS and lysed using radioimmunoprecipitation assay lysis buffer on ice for 15 min. After ultrasonic concussion, the cell lysates were separated by centrifugation at 15,000 × *g* at 4 °C for 15 min. Total protein concentration was quantified using the bicinchoninic acid assay (Fdbio Science).

For western blot analysis, proteins were separated by sodium dodecyl sulfate-polyacrylamide gel electrophoresis and transferred onto polyvinylidene fluoride membranes (Merck Millipore). After blocking with 5% bovine serum albumin (BSA) for 1 h, the membranes were incubated overnight at 4 °C with specific primary antibodies, as indicated in Supplementary Table 3.

### Co-immunoprecipitation (Co-IP) and mass spectrometry analysis

The cells were transfected with plasmids with Flag label. Afterward, cells were lysed with IP cell lysis buffer (Beyotime) at 4 °C for 30 min. Cell lysates were then incubated with anti-Flag antibody (Sigma-Aldrich) overnight at 4 °C. Protein A/G agarose beads (Santa Cruz Biotechnology) were added to the samples and incubated at 4 °C for 4 h. The immune complexes were subsequently washed three times with PBS and eluted with 2× SDS loading buffer (Invitrogen). The eluates were subjected to western blot analysis using the indicated antibodies. Liquid chromatography-mass spectrometry (LC-MS) analysis was performed by Wininnovate Bio (Shenzhen, China).

### In vitro ubiquitination assay

Cells were transfected with siRNA alongside HA-ubiquitin plasmid. After 48 h of transfection, 20 μM MG-132 (MedChemExpress) was added to inhibit proteasomal degradation. Immune complexes were obtained by immunoprecipitation with anti-HA antibody (Dia-An Biotech). The ubiquitination of the designated substrate was then detected via western blot analysis using the indicated antibodies.

### Immunofluorescence (IF)

NPC cells were seeded on slides overnight, then fixed with 4% paraformaldehyde and permeabilised with 0.5% Triton X-100. Subsequently, the cells were blocked with 5% BSA and incubated with indicated primary antibodies overnight at 4 °C. The cells were then stained with fluorophore-conjugated secondary antibodies (Proteintech) in the dark, and the nuclei were counterstained with DAPI (LEAGENE). Fluorescent images were captured using a confocal microscope (Zeiss LSM 980).

### Cycloheximide (CHX) assay

Cells were cultured in six-well plates and transfected with siRNA in advance. After 48 h, the cells were treated with 25 μg/mL CHX (Sigma-Aldrich). Following treatment for 0, 6, 12, and 24 h, the cells were harvested and lysed. Subsequently, the protein abundance was assessed by performing western blot analysis using the indicated antibodies.

### Protein-binding model

Molecular docking calculations were performed using MDockPP online webserver (https://zougrouptoolkit.missouri.edu/MDockPP). The initial protein structures of FASN and USP14 were predicted by AlphaFold2 and obtained from the Uniprot website (https://www.uniprot.org). The protein identifier is AF-P54578-F1 and AF-P49327-F1 for FASN and USP14, respectively. The FASN uiquitination sites were identified using hCKSAAP_UbSite for further analysis of docking conformation [33].

### Xenograft tumour models

Male BALB/c nude mice (3 weeks old, *n* = 36) were obtained from Guangdong Medical Laboratory Animal Center. The mice were randomly assigned to different groups with no blinding. To establish murine xenograft tumours, cells were subcutaneously injected into the right posterior flank of the nude mice. Once the tumours reached a size of 150–200 mm^3^, tumour-bearing mice were either treated with IR (6 Gy) targeted at the tumour site or administered sorafenib (60 mg/kg) intragastrically. The tumours development in the mice were monitored by measuring tumour volume, calculated using the following formula: (width^2^ × length)/2. At the end of the treatment, the mice were euthanised, and the tumour tissues were collected for subsequent immunostaining analyses.

### Clinical sample collection

Paraffin-embedded specimens from 36 newly diagnosed NPC patients and 20 recurrent NPC patients were generously provided by the Sun Yat‐sen University Cancer Center, Guangzhou, China. The biopsy tissues were pathologically confirmed as NPC. The collected samples were utilised for staining experiments and analysis of clinical characteristics in correlation with the available clinical data.

### Immunohistochemical (IHC) staining

The tissue slides were deparaffinised in xylene and gradually rehydrated using an alcohol gradient. Endogenous peroxidase activity was quenched using 3% hydrogen peroxide, followed by antigen retrieval through steaming with a 0.1 M sodium citrate solution (pH 6.0). Subsequently, the sections were blocked with 5% BSA and incubated with the respective primary antibodies overnight at 4 °C. Afterward, the slides were stained with a secondary antibody and visualised using the GTVisionTM III Detection System (GeneTech). Images were captured using the automated microscope (Olympus BX63), and staining scores were evaluated based on staining area and intensity.

### Statistics

The data were analysed using GraphPad Prism (version 9.0) and are presented as the mean ± SEM. Unless otherwise specified, each experiment was independently conducted in triplicate, and the value of ‘*n*’ is indicated in the figure legends. Student’s *t* test (two-tailed) was performed to compare differences between two groups, while one-way analysis of variance (ANOVA) was used for multiple group comparisons. Spearman’s bivariate correlation analysis was utilised to calculate the correlations. Kaplan–Meier analysis was employed to estimate overall survival and progression-free survival.

## Results

### IR induces lipid peroxidation and ferroptosis in NPC cells

**Fig. 1:**
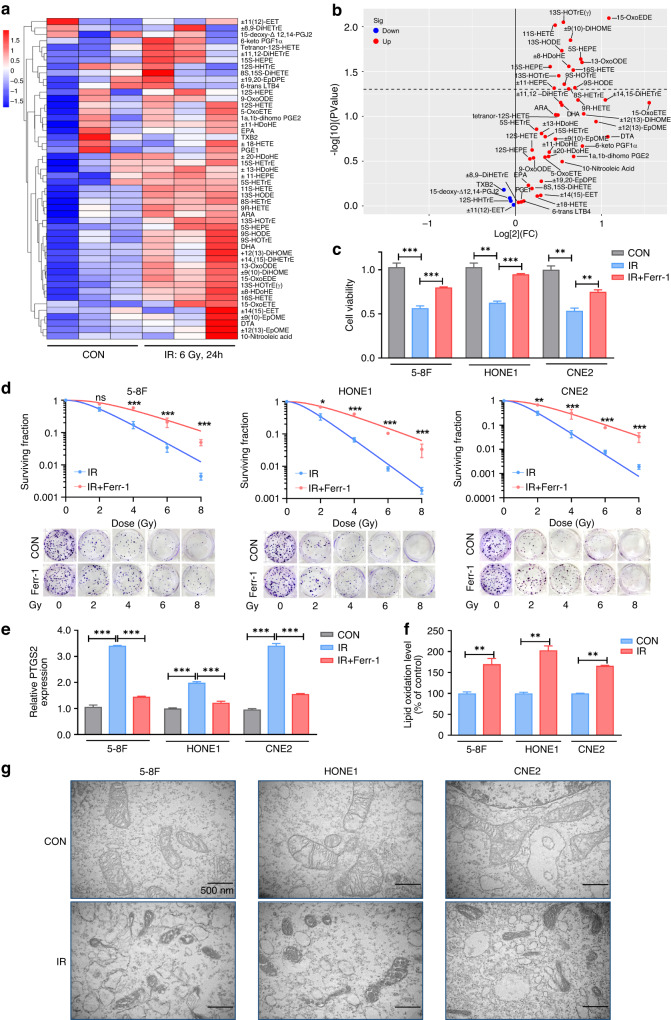
IR induces lipid peroxidation and ferroptosis in NPC cells. **a** Heatmap clustering of oxylipins in NPC cells from the control and IR group. Columns: individual samples; rows: oxylipins; blue: low expression; red: high expression. **b** Volcano plot of the differential oxylipins expression upon IR treatment. **c**, **d** Cell viability assays and clonogenic assays in 5-8 F, CNE2, and HONE1 cells pretreated with ferrostatin-1 or DMEM for 24 h before exposure to 6 Gy IR. **e** NPC cell lines were pretreated with ferrostatin-1 or DMEM for 24 h, followed by exposure to 6 Gy IR. The relative *PTGS2* expression was assessed by qRT-PCR analysis. **f**, **g** The lipid peroxidation (**f**) and morphological changes of mitochondria (**g**) in NPC cells with or without 6 Gy IR exposure. The lipid peroxidation levels were determined using C11-BODIPY 581/591 fluorescence staining. The morphological changes of mitochondria were assessed via TEM. Scale bars: 500 nm. Data are presented as mean ± SEM, *n* = 3 independent repeats. *p* values were calculated using a two-tailed Student’s *t* test. *p < 0.05; **p < 0.01; ***p < 0.001.

Ferroptosis is directly induced by lipid peroxidation, primarily originating from polyunsaturated fatty acid-containing phospholipids [10, 11]. We performed high-throughput targeted metabolomics of oxylipins to determine whether IR affects lipid peroxidation in NPC cells. Compared with control treatment, IR caused a significant increase in oxidised lipids in NPC cells (Fig. 1a, b), particularly the oxidation products of arachidonic acid and linoleic acid (Supplementary Fig. 1A). This observation suggests a potential association between IR and the occurrence of ferroptosis in NPC. Cell viability and clonogenic survival assays on 5-8F, HONE1, and CNE2 cells indicated that IR-mediated cells death could be partially restored by ferrostatin-1, an extensively used ferroptosis antagonist (Fig. 1c, d). The multi-target single-hit model showed that the cells treated with ferrostatin-1 exhibited elevated values for D0, Dq, and SF2 compared to the control group (Supplementary Table 4). This indicates that NPC cells treated with ferrostatin-1 are less susceptible to IR and possess a greater capacity for sublethal damage repair. The expression of prostaglandin endoperoxidase synthase 2 (PTGS2), a marker gene of ferroptosis [12], was upregulated upon IR, and this elevated expression was reversed with ferrostatin-1 treatment (Fig. 1e). C11-BODIPY 581/591 fluorescence staining revealed that lipid peroxidation in NPC cells after IR significantly increased at 24 h and stabilised when exposed to IR doses over 6 Gy (Supplementary Fig. 1B, C). The lipid peroxidation was significantly upregulated by 6 Gy IR exposure after 24 h in NPC cells (Fig. 1f and Supplementary Fig. 1D). Using TEM, we observed that NPC cells exposed to IR exhibited typical features of ferroptosis, including mitochondrial shrinkage, increased membrane density, and thickened cristae (Fig. 1g). Our results collectively indicate that IR triggers ferroptosis in NPC cells.

### GSTM3 facilitates ferroptosis upon IR exposure in vitro

**Fig. 2:**
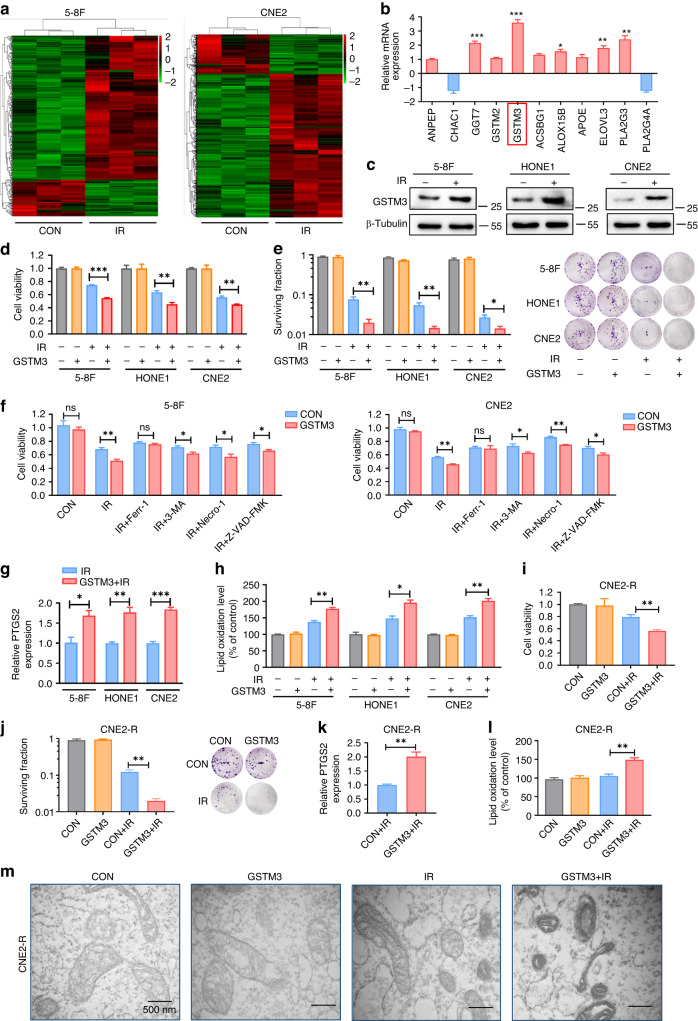
IR-induced ferroptosis is regulated by GSTM3. **a** Heatmap of differentially expressed genes with or without 6 Gy IR exposure in 5-8F and CNE2 cell lines. **b** qRT-PCR analysis of glutathione or lipid metabolism-associated genes after 6 Gy IR exposure. **c** Western blot analysis of GSTM3 protein expression in NPC cell lines at 48 h after 6 Gy IR. **d**, **e** Cell viability assays and clonogenic assays in NPC cells that were transiently transfected with GSTM3 or the empty vector plasmids followed by exposure to 6 Gy IR. **f** The cell viability assays detected the effects of ferrostatin-1, 3-methyladenine, necrostatin-1, and Z-VAD-FMK in NPC cells with GSTM3 overexpression upon IR. **g**, **h** Relative *PTGS2* mRNA expression (**g**) and lipid peroxidation levels (**h**) in empty vector- and GSTM3-overexpressing NPC cells following 6 Gy IR treatment. **i**, **j** Cell viability assays and clonogenic assays in CNE2-R cells transiently transfected with GSTM3 or the empty vector plasmids followed by exposure to 6 Gy IR. **k**, **l** *PTGS2* mRNA expression (**k**) and lipid peroxidation (**l**) in GSTM3-overexpressing or empty vector-transfected CNE2-R cells following 6 Gy IR. **m** TEM images of morphological changes in the mitochondria of CNE2-R cells. Scale bars: 500 nm. Data are presented as mean ± SEM, *n* = 3 independent repeats. *p* values were calculated using the two-tailed Student’s *t* test. *p < 0.05; **p < 0.01; ***p < 0.001.

We performed whole-transcriptome sequencing on 5-8 F and CNE2 cells before and after IR and identified a series of differentially expressed genes (Fig. 2a). Subsequently, we conducted Kyoto Encyclopaedia of Genes and Genomes (KEGG) pathway analysis to determine the biological roles of these differentially expressed genes. Our analysis revealed enrichment of pathways related to lipid metabolism and amino acid metabolism (Supplementary Fig. 2A). As the accumulation of lipoperoxides is considered a hallmark of ferroptosis [10, 11], we identified 107 differentially expressed genes shared by both 5-8F and CNE2 cell lines, including five genes related to glutathione metabolism and six genes related to lipid metabolism (Supplementary Fig. 2B). Among them, the expression of GSTM3 showed the most significant upregulation following IR exposure, as determined by qRT-PCR assay (Fig. 2b). The results revealed that IR substantially increased both mRNA and protein expression of GSTM3 in NPC cell lines (Fig. 2c and Supplementary Fig. 2C, D). GSTM3 expression gradually stabilised following increased IR doses (Supplementary Fig. 2E). These results indicate that GSTM3 may serve as a potential modulator of radiosensitivity in NPC patients.

To further explore the potential role of GSTM3, we constructed NPC cells with overexpression GSTM3 (Supplementary Fig. 2F). Cell viability and clonogenic survival assays showed that GSTM3 overexpression significantly promoted IR-induced cell death (Fig. 2d, e). Remarkably, the enhancement of radiosensitivity by GSTM3 was nearly nullified upon treatment with the ferroptosis inhibitor ferrostatin-1, while the utilisation of the autophagy inhibitor 3-methyladenine, the necroptosis inhibitor necrostatin-1, or the apoptosis inhibitor Z-VAD-FMK did not produce a comparable effect (Fig. 2f). Furthermore, GSTM3 increased the IR-induced PTGS2 expression and lipid peroxidation (Fig. 2g, h and Supplementary Fig. 2G). Conversely, GSTM3 silencing in 5-8F and HONE1 cell lines moderately attenuated IR-induced cell death (Supplementary Fig. 3A, B). IR-induced PTGS2 expression and lipid peroxidation were severely reduced by the silencing of GSTM3 (Supplementary Fig. 3C, D). Collectively, these findings elucidate that GSTM3 is critical for modulating NPC radiosensitivity predominantly by promoting IR-induced ferroptosis.

Subsequently, we generated a radiation-resistant NPC cell line termed CNE2-R and validated its increased resistance to IR in comparison to the parental CNE2 cells (Supplementary Fig. 4A–C). Gamma-H2AX (γ-H2AX), a hallmark of DNA double-stranded breaks, is a critical marker for effectively detecting DNA damage and repair response [34]. Western blot analysis showed a time-dependent increase in γ-H2AX expression following IR in CNE2 cells, whereas CNE2-R cells exhibited a comparatively lower level of γ-H2AX expression (Supplementary Fig. 4D), indicating that CNE2-R cells were less responsive to IR-induced DNA damage and subsequent repair response. Additionally, treatment of CNE2-R cells with ferrostatin-1 did not cause any restoration of cell death induced by IR (Supplementary Fig. 4E, F), and IR did not increase the PTGS2 expression and lipid peroxidation in CNE2-R cells (Supplementary Fig. 4G, H). Furthermore, we observed that the expression of GSTM3 in CNE2-R cells remained unaltered despite exposure to different doses of IR (Supplementary Fig. 4I).

To determine whether GSTM3 mediates IR-induced ferroptosis to alleviate radioresistance in CNE2-R cells, we overexpressed GSTM3 in CNE2-R cells (Supplementary Fig. 4J). GSTM3 overexpression caused decreased cell viability and clonogenic survival upon exposure to IR (Fig. 2i, j). Additionally, upon IR treatment, PTGS2 expression and lipid peroxidation were significantly enhanced in CNE2-R cells overexpressing GSTM3 (Fig. 2k, l and Supplementary Fig. 4K). TEM revealed that IR induced subtle morphological changes in the mitochondria of CNE2-R cells. However, upon GSTM3 overexpression, these mitochondrial changes became more pronounced after IR exposure, characterised by a shrunken shape, increased membrane density, and thickened cristae (Fig. 2m). Overall, our data strongly suggest that GSTM3 effectively alleviates radioresistance by potentiating IR-induced ferroptosis.

### GSTM3 promotes IR-mediated ferroptosis and NPC radiosensitivity in vivo

**Fig. 3:**
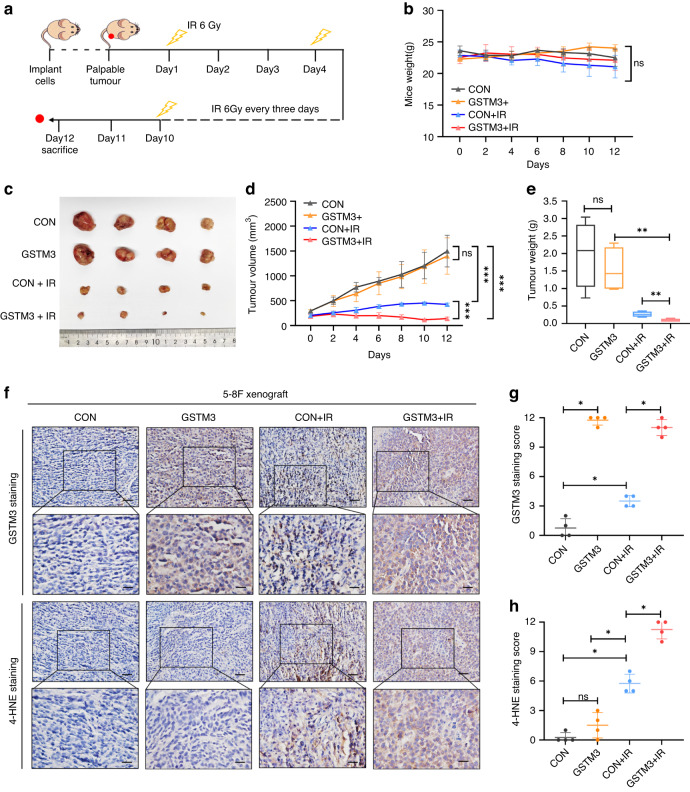
GSTM3 potentiates the radiosensitivity of NPC cells in vivo. **a** Schematic diagram of treatment procedure in subcutaneous tumour xenograft models. The 5-8F cells stably transfected with GSTM3 or empty vector lentivirus were used to construct subcutaneous tumour xenograft models. **b** The weights of the mice from each group were measured every two days. **c**–**e** Macroscopic images (**c**), volume (**d**), and weight (**e**) of the 5-8F xenograft tumours for each group (*n* = 4). **f**–**h** Representative IHC images (**f**) and staining scores of GSTM3 (**g**) and 4-HNE (**h**) of subcutaneous tumours from each group. Scale bars: 50 μm/20 μm. Data are presented as mean ± SEM. *p* values were calculated using two-tailed Student’s *t* test and two-way ANOVA. *p < 0.05; **p < 0.01; ***p < 0.001.

To investigate the impact of GSTM3 on IR-induced ferroptosis in vivo, we subcutaneously injected 5-8F cells with stable GSTM3 overexpression into nude mice, leading to the formation of palpable tumours. Subsequently, the mice bearing xenograft tumours were subjected to regular IR treatments (Fig. 3a). On day 12, the mice were euthanised, and the tumours were dissected for volume measurement and IHC staining. The weights of the mice remained stable throughout the entire treatment period (Fig. 3b). Compared with the control groups, GSTM3 overexpression alone did not exhibit any impact on tumour growth in xenograft models, whereas IR effectively suppressed tumour growth (Fig. 3c–e). Notably, compared to the group treated with IR alone, GSTM3 overexpression combined with IR treatment resulted in a substantial reduction in tumour size and weight (Fig. 3c–e). 4-hydroxy-2-noneal (4-HNE) acts as a ferroptosis marker reflecting the level of lipid peroxidation [35]. IHC staining revealed that IR moderately increased the abundance of GSTM3 and 4-HNE (Fig. 3f–h). Moreover, GSTM3 overexpression combined with IR treatment led to a significantly higher level of the 4-HNE signal (Fig. 3f–h). Collectively, these results suggest that GSTM3 enhances IR-mediated ferroptosis and improves radiosensitivity in NPC.

### GSTM3 stabilises USP14/FASN axis to promote IR-induced ferroptosis

**Fig. 4:**
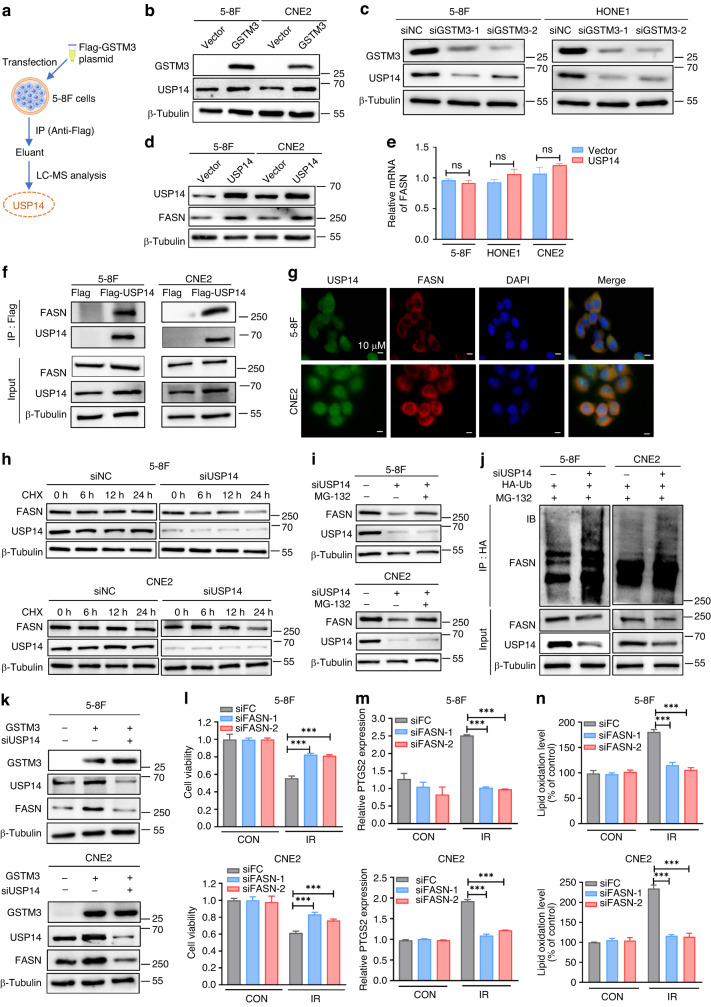
GSTM3 promotes IR-induced ferroptosis partly via the USP14/FASN axis. **a** LC-MS analysis identified USP14 as a potential target of GSTM3. **b**, **c** Western blot analysis of USP14 in NPC cell lines with overexpressed GSTM3 or silencing GSTM3. **d**, **e** The protein expression or mRNA level of FASN in NPC cells transiently transfected with USP14 or the empty vector plasmids. **f** Co-immunoprecipitation assays with anti-Flag antibodies in 5-8F and CNE2 cells revealed that USP14 interacts with FASN in vitro. **g** Immunofluorescence staining revealed the co-localisation of endogenous USP14 (green) and FASN (red) in the cytoplasm. Scale bars: 10 μm. **h** Protein stability of FASN was determined by CHX treatment analysis in NPC cells transfected with siUSP14 or control siRNA. **i** The effect of MG132 in siUSP14 or siNC NPC cell lines. The protein expression of FASN was measured via western blot analysis. **j** NPC cells were transfected with siUSP14 or siNC, as well as HA-Ub plasmid for 48 h. Lysates from cells were immunoprecipitated with anti-HA. Western blot analysis with the indicated antibody was conducted to analyse ubiquitination levels of FASN. **k** Western blot analysis in NPC cells co-transfected with either GSTM3 or the empty vector plasmids alongside siUSP14 or siNC. **l**–**n** 5-8F and CNE2 cells were transfected with either siFASN or control siRNA. The cell viability (**l**), the relative *PTGS2* mRNA level (**m**), and lipid peroxidation level (**n**) were assessed in respective cells after exposure to 6 Gy IR. Data are presented as mean ± SEM, *n* = 3 independent repeats. *p* values were calculated using the two-tailed Student’s *t* test. ***p < 0.001.

To further explore the mechanism underlying how GSTM3 facilitates IR-induced ferroptosis, we conducted LC-MS analysis and identified the deubiquitinase USP14 as a potential target of GSTM3 (Fig. 4a and Supplementary Fig. 5A). Western blot analysis indicated that GSTM3 overexpression effectively increased the protein expression of USP14 (Fig. 4b and Supplementary Fig. 5B), whereas GSTM3 silencing decreased the expression of USP14 (Fig. 4c and Supplementary Fig. 5C). It was proposed that USP14 regulates lipid and carbohydrate metabolism in hepatosteatosis by stabilising FASN, a key lipogenic enzyme [36]. We observed that the protein expression of FASN was correspondingly increased in NPC cells with overexpressing USP14 (Fig. 4d and Supplementary Fig. 5D), whereas silencing USP14 reduced the protein expression of FASN (Supplementary Fig. 5E). However, the mRNA expression of FASN was independent of USP14 overexpression (Fig. 4e), suggesting that USP14 modulates FASN at the posttranscriptional level. Co-IP assays revealed an interaction between USP14 and FASN (Fig. 4f). IF analysis confirmed the co-localisation of USP14 and FASN in the cytoplasm (Fig. 4g). USP14 consists of an N-terminal ubiquitin-like domain (UBL) and a C-terminal catalytic domain (CAT) [37]. FASN is a homodimer comprising seven functional domains [38]. Employing the protein-binding model [33], we observed that USP14^CAT^ was predicted to interact with FASN^TE^ and FASN^MAT^ (Supplementary Fig. 5F).

Furthermore, CHX analysis confirmed that USP14 silencing significantly promoted the degradation of endogenous FASN (Fig. 4h and Supplementary Fig. 5G), suggesting that USP14 can lengthen the half-life of FASN protein. As USP14 is a major regulator of the proteasome and possesses proteasome-associated deubiquitination activity [39], we treated NPC cells with MG132, a proteasome suppressor that impedes ubiquitin degradation. The results showed that silencing USP14-mediated destabilisation of FASN was partly reversed by MG132 (Fig. 4i and Supplementary Fig. 5H). Ubiquitination assays indicated that the polyubiquitination of FASN was increased by USP14 silencing in NPC cells (Fig. 4j), demonstrating that USP14 stabilises the FASN protein by inhibiting its ubiquitin-proteasome degradation in NPC cells. Importantly, western blot analysis showed that FASN and USP14 were upregulated by GSTM3 overexpression, whereas FASN expression was almost completely restored by USP14 silencing (Fig. 4k and Supplementary Fig. 5I). These results indicate that GSTM3 regulates USP14 expression to inhibit the polyubiquitination and subsequent degradation of FASN.

Moreover, cell viability assay showed that FASN silencing resulted in significant restoration of cell viability under IR exposure (Fig. 4l and Supplementary Fig. 6A, B). Furthermore, FASN silencing mitigated IR-induced PTGS2 expression and lipid peroxidation (Fig. 4m, n and Supplementary Fig. 6C), suggesting that FASN mediates IR-induced ferroptosis to improve radiosensitivity in NPC. Collectively, our findings indicate that GSTM3 promotes IR-induced ferroptosis and enhances radiosensitivity through USP14/FASN axis in NPC.

### GSTM3 facilitates IR-induced ferroptosis by suppressing GPX4

To investigate the possibility of additional mechanisms underlying IR-induced ferroptosis promoted by GSTM3, we examined the expressions of several key genes (*GPX4, ACSL3, SLC7A11, TF, FTL*) involved in the ferroptosis pathways [11, 12] in NPC cells with GSTM3 overexpression. Western blot analysis revealed that the overexpression of GSTM3 reduced the expression of GPX4 (Supplementary Fig. 7A), a glutathione peroxidase possessing potent antioxidant activity [40]. Additionally, Co-IP assays revealed an interaction between GSTM3 and GPX4 (Supplementary Fig. 7B). IF staining demonstrated the co-localisation of GSTM3 and GPX4 in the cytoplasm (Supplementary Fig. 7C), indicating that GSTM3 interacts with GPX4 and suppresses its expression. To investigate the potential involvement of GPX4 in IR-induced ferroptosis, we conducted functional experiments in NPC cells overexpressing GPX4 (Supplementary Fig. 7D). Remarkably, the overexpression of GPX4 significantly reversed IR-induced cell death and notably reduced the levels of PTGS2 and lipid peroxidation following IR treatment (Supplementary Fig. 7E–G). These results suggest that GPX4 acts as a downstream target of GSTM3, mediating IR-induced ferroptosis in NPC. Furthermore, in the subcutaneous tumour xenograft model, the expression of USP14 and FASN were increased, while the protein levels of GPX4 were decreased in the group with GSTM3 overexpression (Supplementary Fig. 8A). The schematic diagram illustrating the mechanisms by which GSTM3 enhances IR-induced ferroptosis in NPC was presented in Supplementary Fig. 8B.

### FINs and IR synergistically trigger ferroptosis and sensitise cancer cells to radiotherapy

**Fig. 5:**
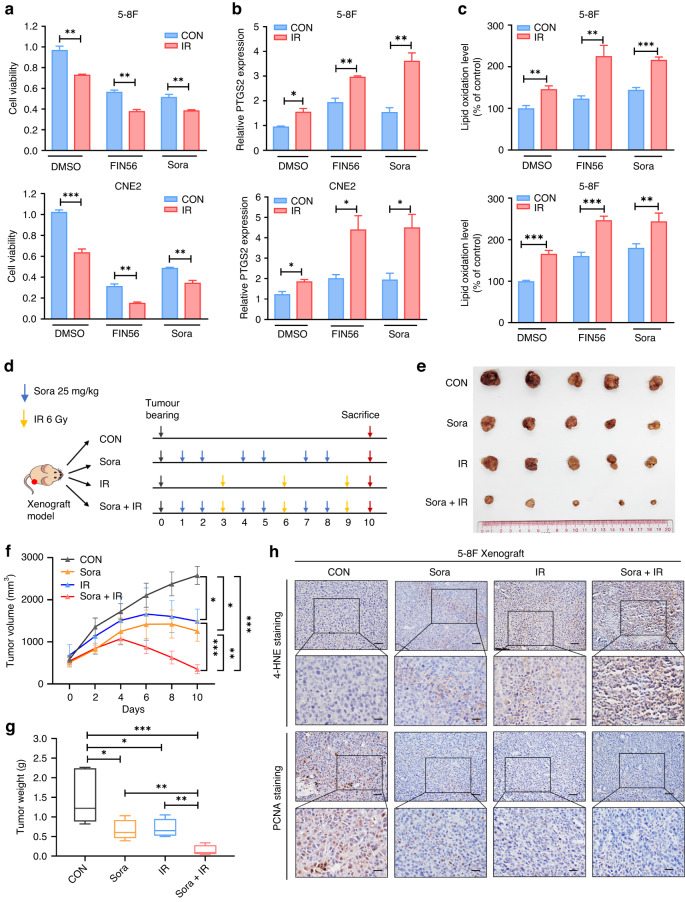
FINs sensitise cancer cells to radiotherapy in vitro and in vivo. **a**–**c** 5-8F and CNE2 cell lines were pretreated with FIN56, sorafenib, or DMEM for 24 h before 6 Gy IR exposure. The cell viability (**a**), relative *PTGS2* expression (**b**), and lipid peroxidation levels (**c**) were measured in the respective cell lines. **d** The therapeutic modality in the mice with 5-8F subcutaneously xenografted tumours. The mice with xenografts were treated with 6 Gy IR or intragastric administration of sorafenib. **e**–**g** The size (**e**), volume (**f**), and weight (**g**) of the xenograft tumours in control, sorafenib, IR, or combination therapy groups (*n* = 5). **h** IHC staining images of 4-HNE and PCNA of xenograft tumours from each group. Scale bars: 50 μm/20 μm. Data are presented as mean ± SEM. *p* values were calculated using two-tailed Student’s *t* test and two-way ANOVA. *p < 0.05; **p < 0.01; ***p < 0.001.

Ferroptosis can be induced by different types of FINs [16, 17], prompting us to investigate whether the combined treatment of FINs and IR could synergistically potentiate ferroptosis and enhance NPC radiosensitivity. In this study, the FINs sorafenib and FIN56, which inhibit SLC7A11 and GPX4 respectively, were used to explore the potential role in NPC. Individual treatment with FINs or IR suppressed the NPC cells viability and promoted PTGS2 expression and lipid peroxidation level (Fig. 5a–c). Notably, the combined treatment with FINs and IR resulted in a dramatic increase in NPC cells death, the PTGS2 expression, and lipid peroxidation (Fig. 5a–c). Considering the selectivity of sorafenib as a ferroptosis inducer for certain tumour cell lines [41], we conducted further investigations to assess its cytotoxic effects in NPC cell lines. The cell viability assays revealed that sorafenib induced cell death in 5-8F and CNE2, while ferrostatin-1 rescued the lethal effects upon sorafenib treatment (Supplementary Fig. 9A). C11-BODIPY fluorescence staining indicated an elevation in lipid peroxidation levels induced by sorafenib, which could be counteracted by ferrostatin-1 (Supplementary Fig. 9B). Moreover, the synergistic effect of sorafenib and IR was mitigated in the presence of ferrostatin-1 treatment (Supplementary Fig. 9A, B). Given that sorafenib is a multiple-target tyrosine kinase inhibitor, we further investigated the synergy between IR and downregulation of SLC7A11. The results showed that the downregulation of SLC7A11 significantly promoted IR-induced cell death and increased lipid peroxidation following IR treatment (Supplementary Fig. 9C–E).

Subsequently, we generated subcutaneous tumour xenograft models to assess the combined effect of sorafenib and IR on NPC radiosensitivity in vivo (Fig. 5d). The results indicated that the individual treatment with sorafenib or IR reduced the volume and weight of tumours (Fig. 5e–g). Significantly, the combined treatment of sorafenib and IR exhibited substantial suppression of xenograft growth (Fig. 5e–g). Sorafenib and IR increased the expression of 4-HNE, a hallmark of ferroptosis, and decreased the expression of proliferating cell nuclear antigen (PCNA), a biomarker of proliferation (Fig. 5h). Notably, the combination of sorafenib with IR treatment further enhanced 4-HNE expression and suppressed PCNA expression (Fig. 5h). These findings reveal that the combined treatment of FINs and IR synergistically potentiates ferroptosis in NPC cells, resulting in a significant sensitisation of cancer cells to radiotherapy.

### Low expression of GSTM3 is correlated with tumour relapse and poor prognosis in NPC

**Fig. 6:**
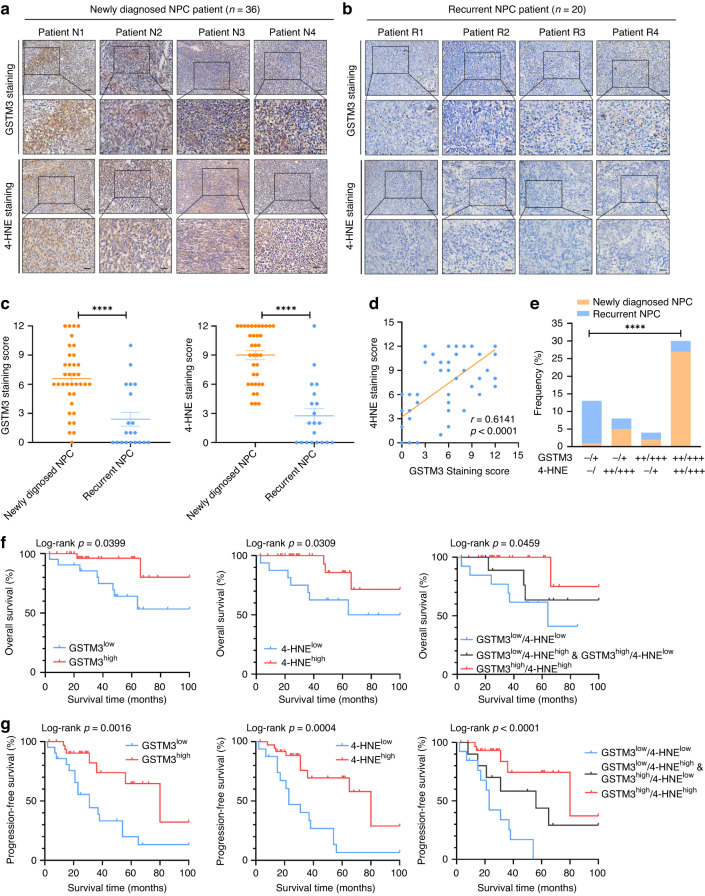
Low expression of GSTM3 is correlated with tumour relapse and poor prognosis in NPC. **a**, **b** Representative IHC images of GSTM3 and 4-HNE staining in patients with newly-diagnosed NPC (**a**) or recurrent NPC (**b**). Scale bars: 50 μm/20 μm (inset). **c** IHC scoring of GSTM3 and 4-HNE staining in patients with newly-diagnosed NPC or recurrent NPC. **d** Correlations between the staining scores of GSTM3 and 4-HNE. **e** The proportion of recurrence status in patients with GSTM3 and 4-HNE expression as detected by IHC. **f**, **g** Kaplan–Meier analysis of overall survival and progression-free survival grouping by GSTM3 and 4-HNE expression levels. Data are presented as mean ± SEM. *p* values were calculated using two-tailed Student’s *t* test. ****p < 0.0001.

To evaluate the clinical significance of GSTM3 in radiotherapy response and prognosis, we collected tumour tissues from 36 newly diagnosed NPC patients and 20 recurrent NPC patients. Remarkably, the newly diagnosed NPC samples exhibited strongly positive staining for GSTM3 and 4-HNE (Fig. 6a), while the recurrent tumour tissues showed relatively negative expression of GSTM3 and 4-HNE (Fig. 6b). The association between the clinical characteristics and the expression of GSTM3 and 4-HNE in NPC patients is presented in Supplementary Tables 5 and 6, respectively. These results indicated that tumour relapse was associated with weak expression of GSTM3 and 4-HNE in NPC patients (Fig. 6c and Supplementary Fig. 10A, B). Moreover, there is a positive association between GSTM3 and 4-HNE staining (Fig. 6d). A moderate or strong positive dual staining signal of GSTM3 and 4-HNE was correlated with low incidence rates of NPC recurrence (Fig. 6e). Kaplan–Meier analysis revealed that a low expression of either GSTM3 or 4-HNE was associated with poor overall survival and progression-free survival in NPC patients (Fig. 6f, g). Furthermore, the simultaneous low expressions of both GSTM3 and 4-HNE conferred a higher risk of disease progression and mortality (Fig. 6f, g). Based on The Cancer Genome Atlas, we found that high GSTM3 expression was correlated with longer disease-free survival (Supplementary Fig. 10C). These findings suggest that GSTM3 contributes to the radiotherapy response associated with ferroptosis, predicting a favourable prognosis in NPC patients.

## Discussion

Radiotherapy is a standard treatment modality for patients diagnosed with NPC [2]. However, some patients suffer from residual neoplasms and local recurrence due to radioresistance [5]. NPC radioresistance is attributed to various regulatory mechanisms, including DNA damage response signalling, cancer stem cell phenotype, abnormal cell-cycle progression, hypoxic properties, and the tumour immune microenvironment [42–45]. However, our current understanding of these mechanisms has not fully addressed the challenges of radioresistance and treatment failure. Consequently, it is crucial to explore the underlying mechanisms of radioresistance to identify potential strategies for improving therapeutic response.

Ferroptosis plays a critical role in mediating radiation response in hepatocellular carcinoma, lung cancer, and melanoma [12–14]. Studies have reported that Epstein–Barr virus (EBV) infection-induced GPX4 can enhance chemoresistance and promote tumour progression by inhibiting ferroptosis [46]. Cephalosporin, itraconazole, and cucurbitacin B have been shown to trigger ferroptosis to exert potential antitumour effects in NPC [20–23]. However, the impact of ferroptosis on NPC radiosensitivity remains unclear. Ferroptosis is characterised by the excessive peroxidation of polyunsaturated fatty acid-containing phospholipids [11]. In this study, we conducted a targeted metabolomic analysis of oxylipins in NPC cells and found that IR resulted in an increase in lipid peroxidation, specifically the oxidation products of arachidonic acid and linoleic acid. Importantly, we demonstrated that IR-induced cell death could be rescued by the ferroptosis inhibitor ferrostatin-1. Furthermore, IR exposure resulted in an increase of PTGS2 expression, accumulation of lipid peroxidation, and characteristic morphological changes in NPC cells, collectively suggesting that ferroptosis potentially plays a crucial role in regulating the sensitivity of NPC to radiotherapy.

GSTM3 participates in the intricate processes of tumourigenesis and the progression in various malignancies. GSTM3 reportedly predicts high susceptibility, tumour malignant behaviours, and poor prognosis in some cancer types [28–32]. However, GSTM3 acts as a tumour suppressor to inhibit tumourigenesis in gastric cancer, alleviate the aggressiveness in renal cell carcinoma, and alter the malignant metabolic pattern in pancreatic cancer [25, 26, 47]. Moreover, GSTM3 reverses the radioresistance through cell cycle arrest and apoptosis facilitation in hepatocellular carcinoma [27]. However, the functions and mechanisms of GSTM3 in NPC have not been investigated. Our study revealed that GSTM3 was upregulated by IR and sensitised NPC cells to radiotherapy by potentiating IR-mediated ferroptosis. Additionally, GSTM3 alleviated the radioresistance in radiation-resistant NPC cells. These findings suggest that GSTM3 holds promise as a potential biomarker for promoting radiotherapy sensitivity in NPC. While the prognostic value of GSTM3 has been reported in various tumours, its potential as a prognostic indicator in NPC remains uncertain. In our study, we observed that a low GSTM3 expression conferred a higher risk of locoregional recurrence and predicted poor overall survival and progression-free survival in NPC patients. Our findings indicate that GSTM3 serves as a prognostic indicator for NPC, laying the foundation for exploring potential treatment strategies for patients with radiation-resistant or recurrent NPC.

USP14, a major deubiquitinase reversibly associated with the proteasome, participates in IR-induced DNA double-strand breaks repair via non-homologous end joining [48]. Moreover, USP14 is involved in regulating ferroptosis, autophagy, amino acid metabolism, and immune suppression to mediate tumour progression and treatment response [49, 50]. In this study, we found that GSTM3 stabilised the expression of deubiquitinase USP14, thereby inhibiting the ubiquitination and subsequent degradation of FASN. As a pivotal enzyme involved in the lipid biosynthesis pathway, FASN may supply polyunsaturated fatty-acid for the production of lipid peroxidase. O-GlcNAcylation enhances the transcriptional activity of FASN to facilitate ferroptosis in mesenchymal pancreatic cancer [51]. FASN is related to Alzheimer’s disease-related toxicity that modulates lipid peroxidation and induces ferroptosis [52]. However, FASN remodels oxidised phospholipids to escape ferroptosis in KRAS-mutant lung cancer [53]. In our study, we demonstrated that GSTM3 regulates USP14/FASN axis to potentiate IR-induced ferroptosis in NPC, presumably by enhancing the synthesis of lipid peroxidation.

As a glutathione peroxidase, GPX4 converts lipid hydroperoxides into lipid alcohols, ultimately eliminating lipid peroxidation [40]. GPX4 inhibition-mediated ferroptosis is essential for the radiosensitivity of breast cancer and hepatocellular carcinoma [54, 55]. Additionally, ferroptosis resulting from GPX4 deficiency is associated with antitumour immunity, malignant biological properties, and platinum drugs resistance [56, 57]. EBV infection-induced GPX4 reduces the sensitivity of cells to ferroptosis via p62-Keap1-NRF2 signalling pathway, leading to chemoresistance and tumour progression [46]. Cucurbitacin B and lupeol initiate the mechanism of ferroptosis in NPC by downregulating the expression of GPX4 [23, 58]. Our findings reveal that GPX4 serves as a downstream effector of GSTM3 to regulate IR-induced ferroptosis and NPC radiosensitivity.

FINs exert significant effects on improving radiotherapy sensitivity and enhancing the immunotherapy response [12, 17]. Numerous studies have demonstrated that sorafenib showed potent induction of ferroptosis in various cancers, unravelling novel mechanisms through which sorafenib induced this process [35, 59, 60]. However, the status of sorafenib as one of the FIN has recently been challenged [41]. The selectivity of sorafenib as a ferroptosis inducer in specific cell lines and the intricate molecular mechanisms involved remain largely unknown. Intriguingly, we found that sorafenib combined with IR synergistically triggered ferroptosis and exhibited significant radiosensitising effects in NPC. These findings hold promise in overcoming the challenge of radiotherapy resistance in NPC and may provide clinicians with new strategies to improve treatment outcomes. Although SLC7A11 was not regulated by GSTM3, the targeting of SLC7A11 by sorafenib substantially promoted radiotherapy sensitisation, implying that other critical pathways parallel to GSTM3 might mediate IR-induced ferroptosis.

Overall, this study revealed the significant role of GSTM3 in IR-induced ferroptosis and radiotherapy sensitivity in NPC. It highlighted the involvement of GSTM3 in stabilising the USP14/FASN axis and targeting GPX4 as key mechanisms underlying these processes. Combining IR treatment with ferroptosis inducers synergistically improved NPC radiosensitivity and suppressed tumour growth. Notably, a decrease in GSTM3 abundance predicted tumour relapse and poor prognosis. These findings provide valuable insights for the development of promising treatment strategies targeting radiation-resistant or recurrent NPC.

### Supplementary information

Supplementary Data

## Data Availability

The datasets used and/or analysed in the current study are available from the corresponding author on reasonable request.
