# Supplementary material for: GSTM3 enhances radiosensitivity of nasopharyngeal carcinoma by promoting radiation-induced ferroptosis through USP14/FASN axis and GPX4
Source: Br J Cancer. 2024 Jan 16;130(5):755–68. doi: 10.1038/s41416-024-02574-1 (PMC10912431; doi:10.1038/s41416-024-02574-1)
Supplement: Supplementary file 1 — Supplementary Data [file 41416_2024_2574_MOESM1_ESM.docx]

**Supplementary Information**

**GSTM3 enhances radiosensitivity of nasopharyngeal carcinoma by promoting radiation-induced ferroptosis through USP14/FASN axis and GPX4**

**Supplementary Figures:**

Supplementary Fig 1. IR triggers lipid peroxidation and ferroptosis.

Supplementary Fig 2. GSTM3 is increased upon IR exposure and facilitates ferroptosis.

Supplementary Fig 3. GSTM3 silencing suppresses IR-induced ferroptosis.

Supplementary Fig 4. GSTM3 promotes IR-induced ferroptosis in CNE2-R.

Supplementary Fig 5. USP14 inhibits the polyubiquitination and degradation of FASN.

Supplementary Fig 6. FASN mediates IR-induced ferroptosis to improve radiosensitivity in NPC.

Supplementary Fig 7. GPX4 acts as a target of GSTM3 to regulate IR-induced ferroptosis.

Supplementary Fig 8. Proposed mechanism of GSTM3 improving IR-induced ferroptosis in NPC.

Supplementary Fig 9. Sorafenib induces ferroptosis and enhances radiosensitivity by targeting SLC7A11 in NPC.

Supplementary Fig 10. Low GSTM3 and 4-HNE abundance predicts tumor relapse and poor prognosis.

**Supplementary Tables:**

Supplementary Table 1. List of siRNA sequences.

Supplementary Table 2. List of PCR primers sequences.

Supplementary Table 3. List of antibodies used in this study.

Supplementary Table 4. Radiobiological Parameters of NPC cells in different group.

Supplementary Table 5. Association of the expression of GSTM3 with clinical features in 56 patients with NPC.

Supplementary Table 6. Association of the expression of 4-HNE with clinical features in 56 patients with NPC.


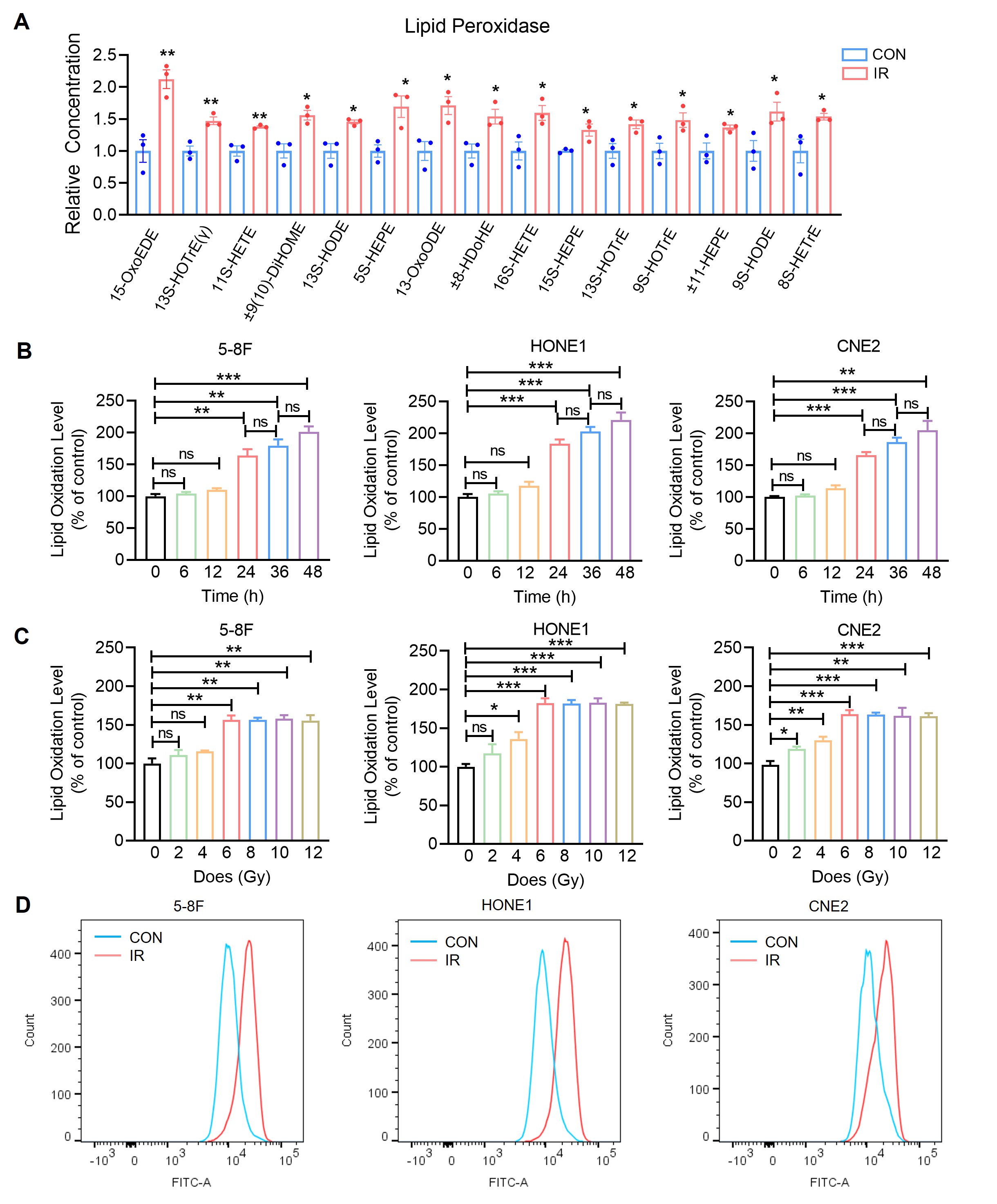


**Supplementary Fig 1.** **IR triggers lipid peroxidation and ferroptosis. A** The relative concentration of lipid peroxidation upon IR induction measured by high-throughput targeted metabolomics of oxylipins. **B**, **C** The lipid peroxidation levels in NPC cells exposed to different IR doses and at different time points were determined using C11-BODIPY 581/591 fluorescence staining. **D** The lipid peroxidation in NPC cells with or without 6 Gy IR exposure after 24 h. Data are presented as the mean ± SEM. Comparisons were made using the Student’s *t*-test. **p* < 0.05; ***p* < 0.01; ****p* < 0.001.

**
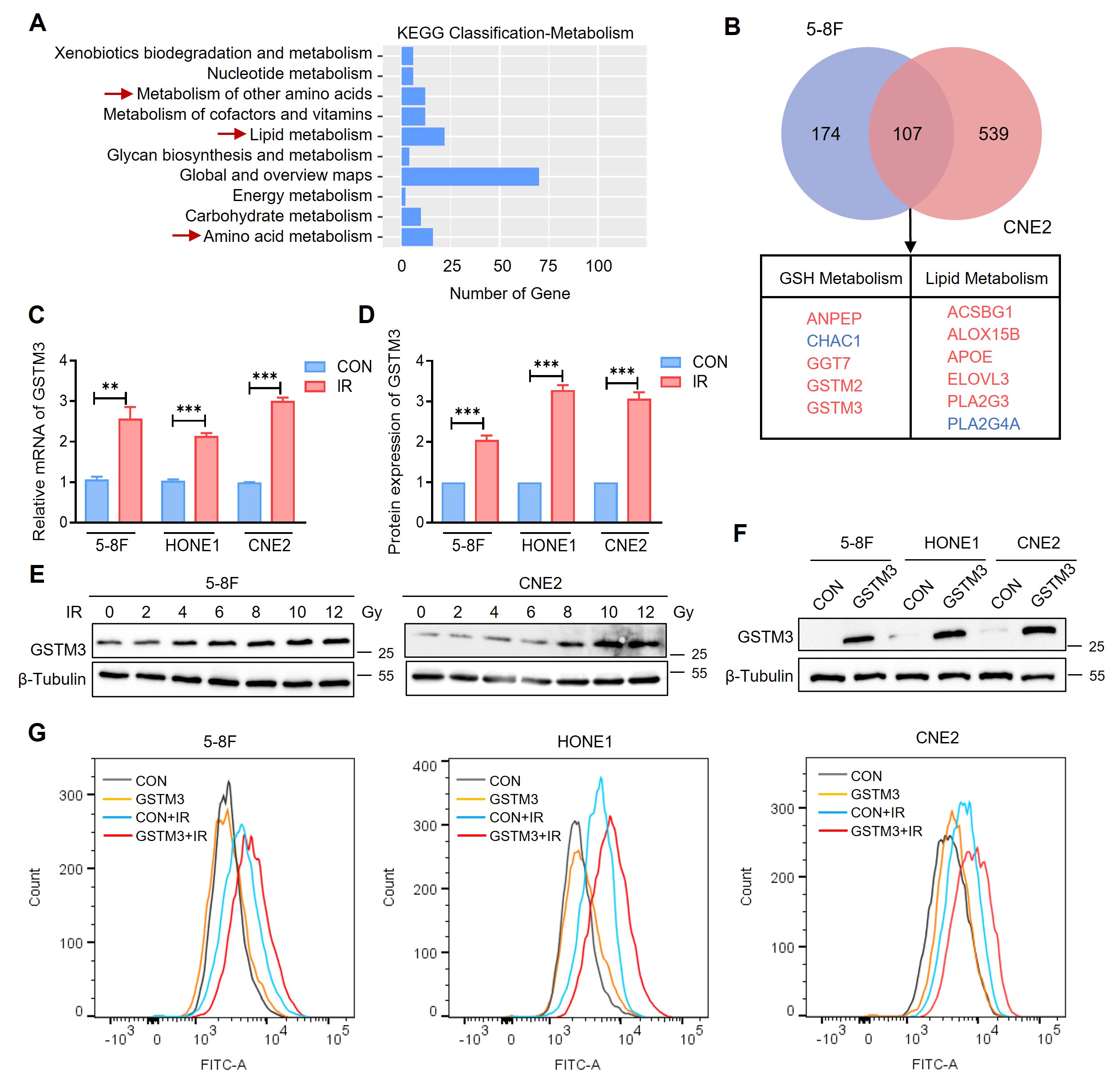
**

**Supplementary Fig 2.** **GSTM3 is increased upon IR exposure and facilitates ferroptosis.** **A** The KEGG analysis identified the metabolism-related pathways in NPC cells following IR exposure. **B** Venn diagram of differentially expressed genes in 5-8F and CNE2 cells. **C, D** The mRNA and protein expression of GSTM3 after 6 Gy IR. **E** IR increased the protein expression of GSTM3 in a dose-dependent manner. **F** Western blot analysis of GSTM3 in NPC cells with overexpressed GSTM3. **G** The lipid peroxidation levels in GSTM3-overexpressing or empty vector–transfected NPC cells followed by exposure to 6 Gy IR. Data are presented as the mean ± SEM. Comparisons were made using the Student’s *t*-test. **p* < 0.05; ***p* < 0.01; ****p* < 0.001.

**
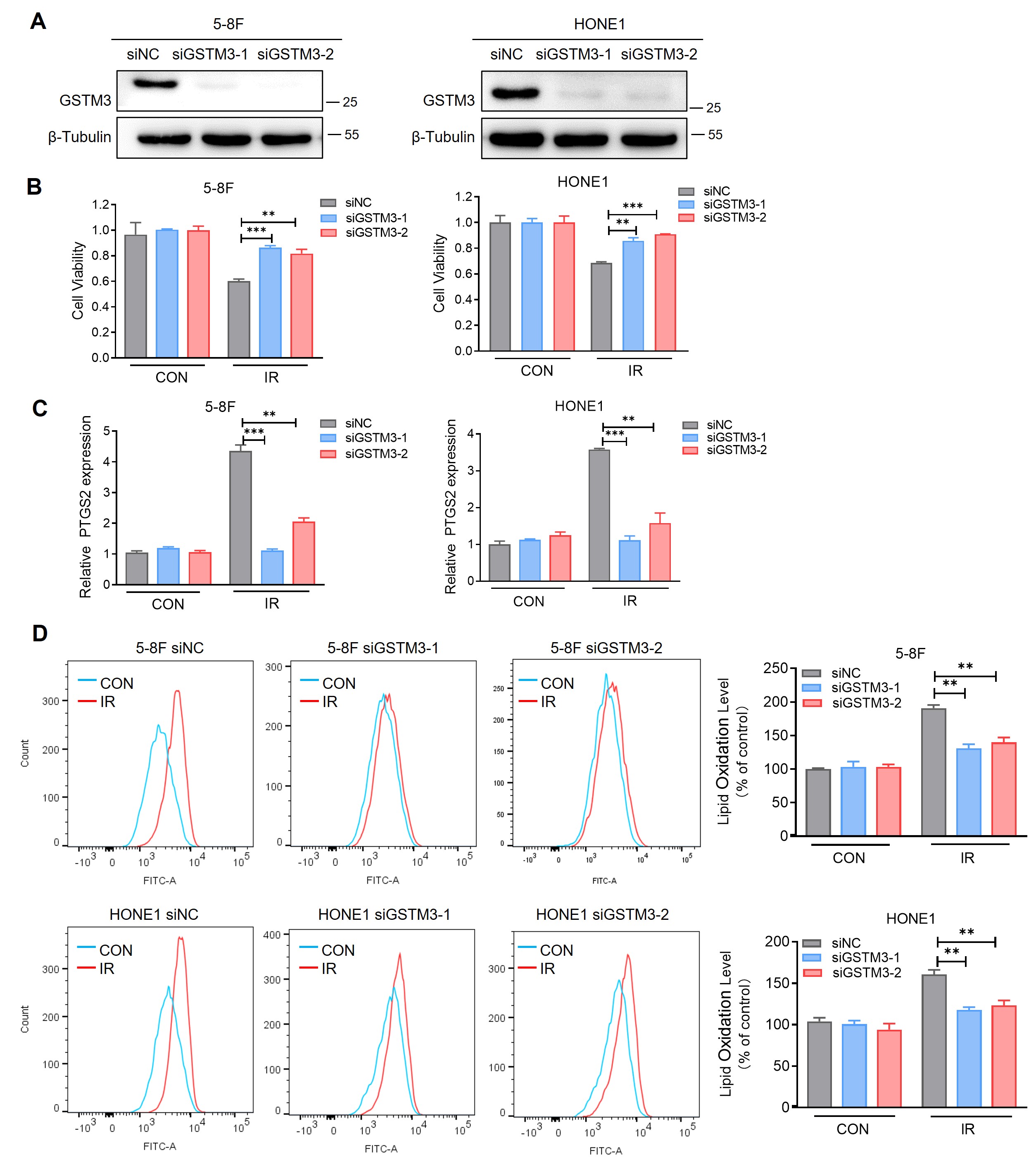
**

**Supplementary Fig 3.** **GSTM3 silencing suppresses IR-induced ferroptosis.** **A** Western blot analysis of GSTM3 in 5-8F and HONE1 cells transfected with siNC or siGSTM3. **B** Cell viability assays in NPC cells transfected with siNC or siGSTM3 followed by 6 Gy IR exposure. **C** The relative *PTGS2* expression upon IR treatment in NPC cells transfected with siNC or siGSTM3. **D** The lipid peroxidation levels in NPC cells transfected with siNC or siGSTM3 after IR treatment. Data are presented as the mean ± SEM. Comparisons were made using the Student’s *t*-test. ***p* < 0.01; ****p* < 0.001.

**
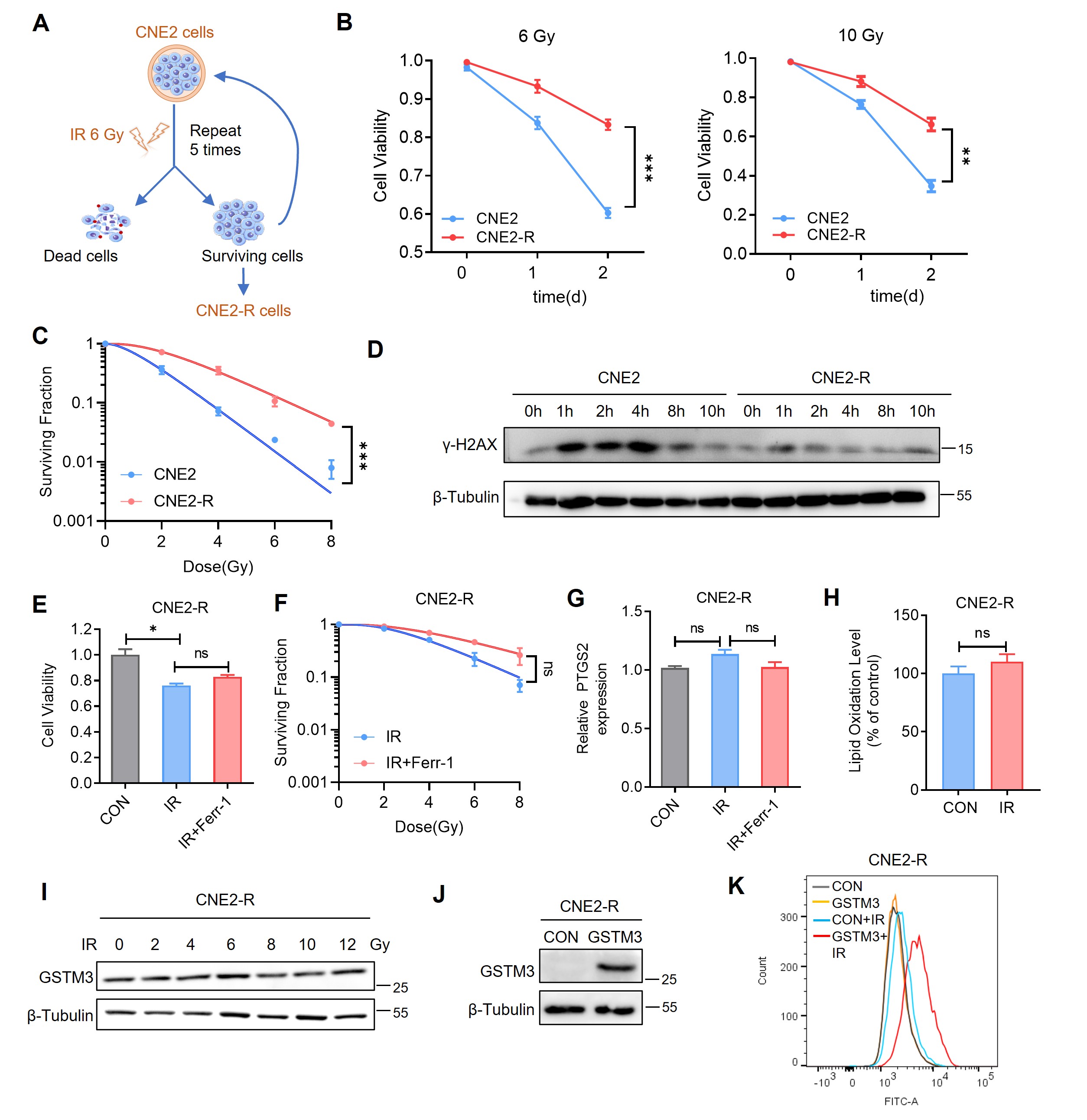
**

**Supplementary Fig 4.** **GSTM3 promotes IR-induced ferroptosis in CNE2-R.**

**A** Schematic representation for establishment of CNE2-R cells. **B-D** Cell viability assays (B), clonogenic assays (C), and γ-H2AX protein expression (D) in CNE2 and CNE2-R cells upon IR induction. **E-G** CNE2-R cells were pretreated with ferrostatin-1 or DMEM for 24 h followed by exposure to 6 Gy IR. Then Cell viability assays (E), clonogenic assays (F), and the relative PTGS2 expression (G) in CNE2-R were measured. **H** The lipid peroxidation levels in CNE2-R cells after exposure to 6 Gy IR. **I** The protein expression of GSTM3 was unaffected by IR. **J** Western blot analysis of GSTM3 in CNE2-R cells transfected with either GSTM3 or control plasmids. **K** The lipid peroxidation levels after exposure to 6 Gy IR in CNE2-R cells with GSTM3 overexpression. Data are presented as the mean ± SEM. Comparisons were made using Student’s *t*-test or one-way ANOVA. **p* < 0.05; ***p* < 0.01; ****p* < 0.001.

**
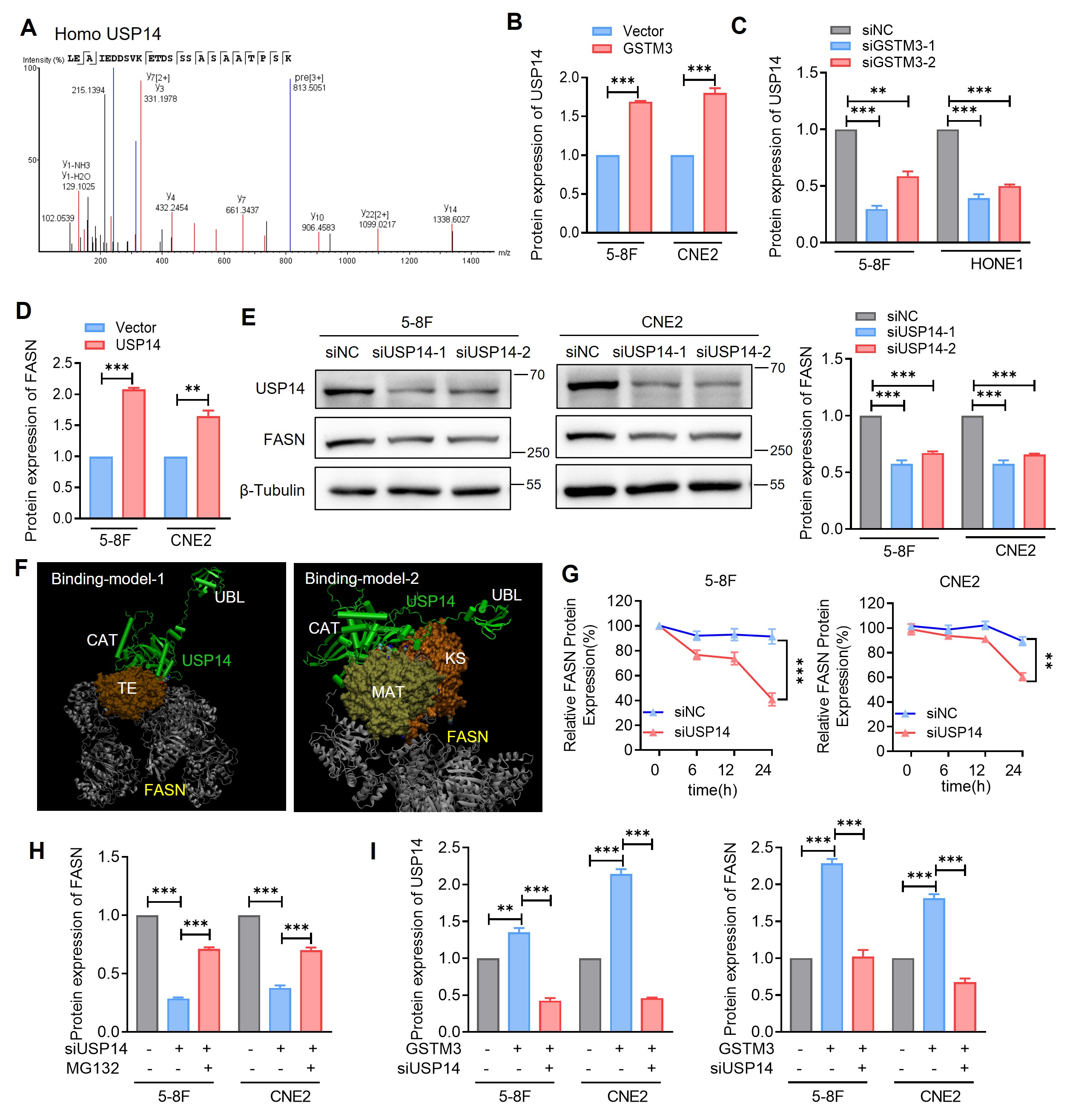
**

**Supplementary Fig 5. USP14 inhibits the polyubiquitination and degradation of FASN.** **A** The mass spectrometry analysis identified GSTM3 junction specific peptides of USP14. **B, C** Western blot analysis of USP14 protein in NPC cells with overexpressed GSTM3 or silencing GSTM3. **D, E** The FASN protein expression in NPC cells transfected with USP14 overexpression plasmid or siUSP14. **F** The binding domains of USP14 and FASN were forecasted by the binding model. **G** Protein stability of FASN was determined through CHX analysis in NPC cells transfected with either siUSP14 or siNC. **H** The protein expression of FASN in siUSP14-transfected NPC cells with or without MG132 treatment. **I** The protein expression of USP14 and FASN in NPC cells co-transfected with GSTM3 plasmid alongside siUSP14. Data are presented as the mean ± SEM. Comparisons were made using Student’s *t*-test or one-way ANOVA. **p* < 0.05; ***p* < 0.01; ****p* < 0.001.

**
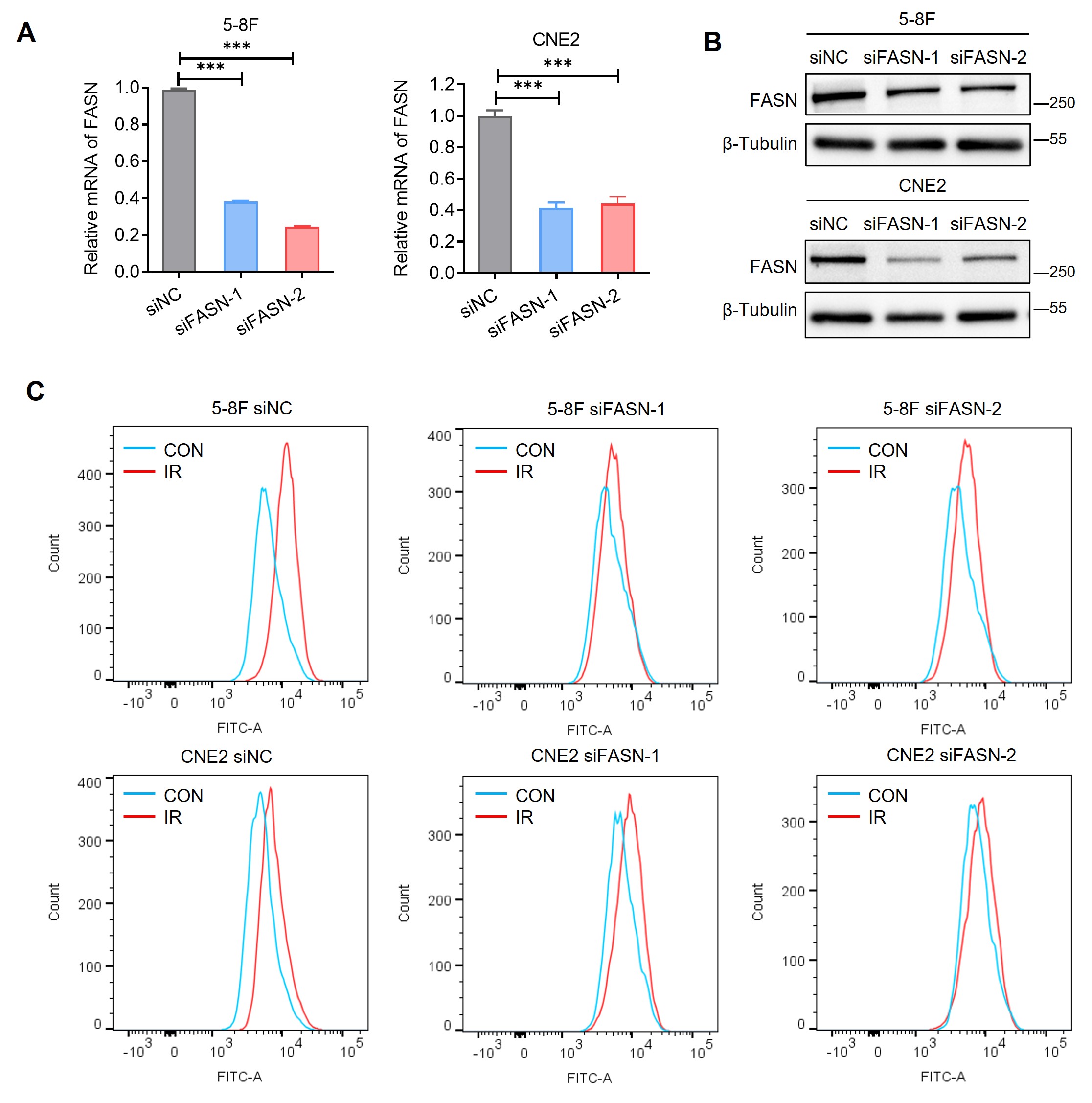
**

**Supplementary Fig 6. FASN mediates IR-induced ferroptosis to improve radiosensitivity in NPC. A** qRT-PCR assessed the relative FASN mRNA expression in 5-8F and CNE2 cells transfected with siFASN or control siRNA. **B** Western blot analysis of FASN protein expression in siNC- or siFASN- NPC cells. **C** 5-8F and CNE2 cells transfected with siFASN or siNC were exposure to 6 Gy IR. After 24 h, the lipid peroxidation levels were measured via C11-BODIPY 581/591 fluorescence staining. Data are presented as the mean ± SEM. Comparisons were made using the two-tailed Student’s *t*-test. ****p* < 0.001.

**
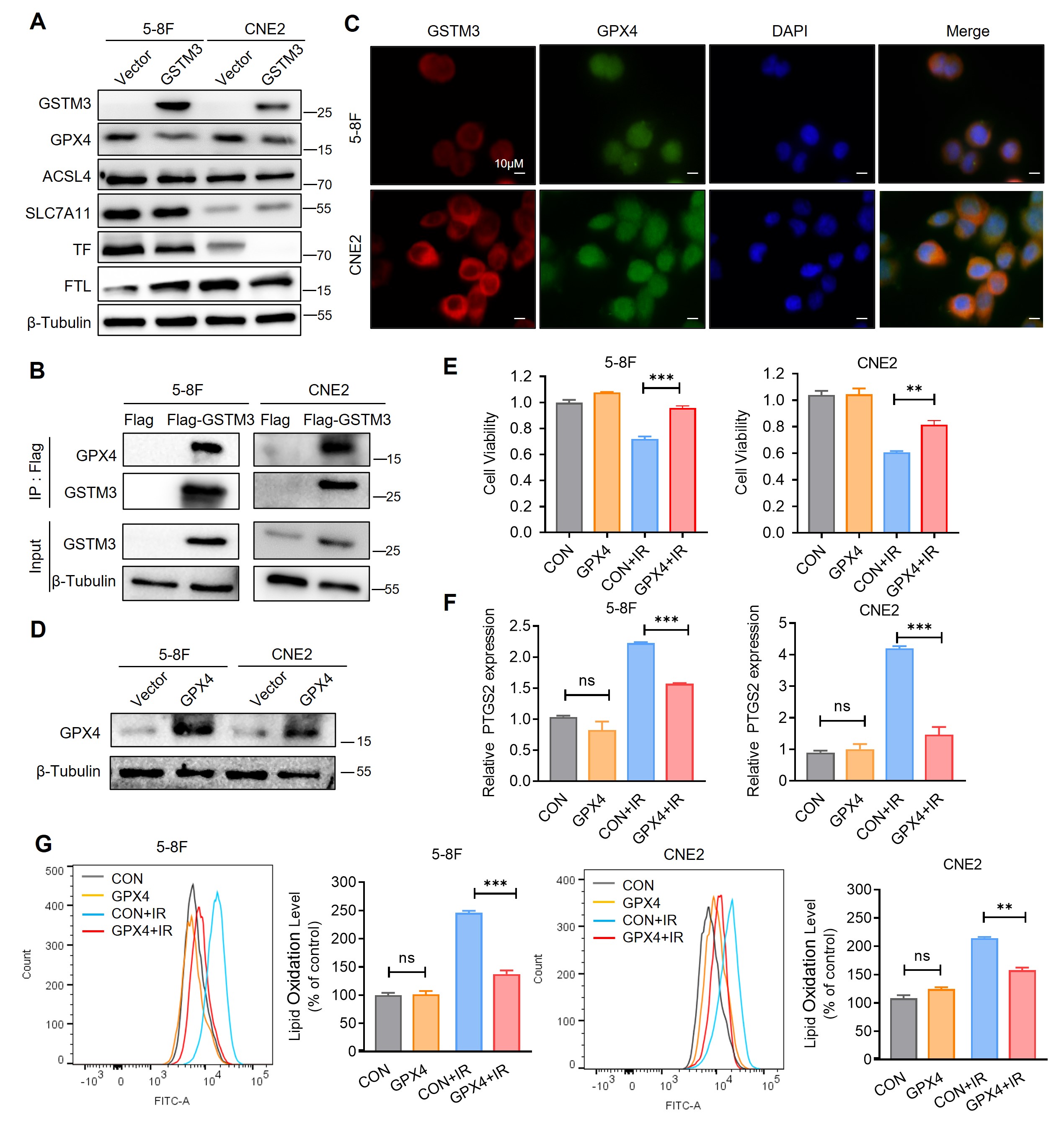
**

**Supplementary Fig 7.** **GPX4 acts as a target of GSTM3 to** **regulate IR-induced ferroptosis.** **A** Western blot analysis of ferroptosis-related proteins in 5-8F and CNE2 cells transfected with GSTM3 plasmid. **B** Co-immunoprecipitation assays with anti-Flag antibodies in 5-8F and CNE2 cells revealed the interaction between endogenous GSTM3 and GPX4. **C** Immunofluorescence staining revealed the co-localization of endogenous GSTM3 (red) and GPX4 (green) in the cytoplasm. Scale bars: 10 μm. **D** Western blot analysis of GPX4 protein in 5-8F and CNE2 cells with GPX4 overexpression or empty vector plasmids. **E-G** 5-8F and CNE2 cells were transiently transfected with overexpressed GPX4 or empty vector plasmids. After 24 h, the cells were pretreated with 6 Gy IR. Then the cell viability (E) in each group was determined using Cell Counting Kit-8. The relative *PTGS2* mRNA levels (F) were measured via qRT-PCR. The lipid peroxidation levels (G) were assessed via C11-BODIPY 581/591 staining. Data are presented as the mean ± SEM. Comparisons were made using the two-tailed Student’s *t*-test. ***p* < 0.01; ****p* < 0.001.

**
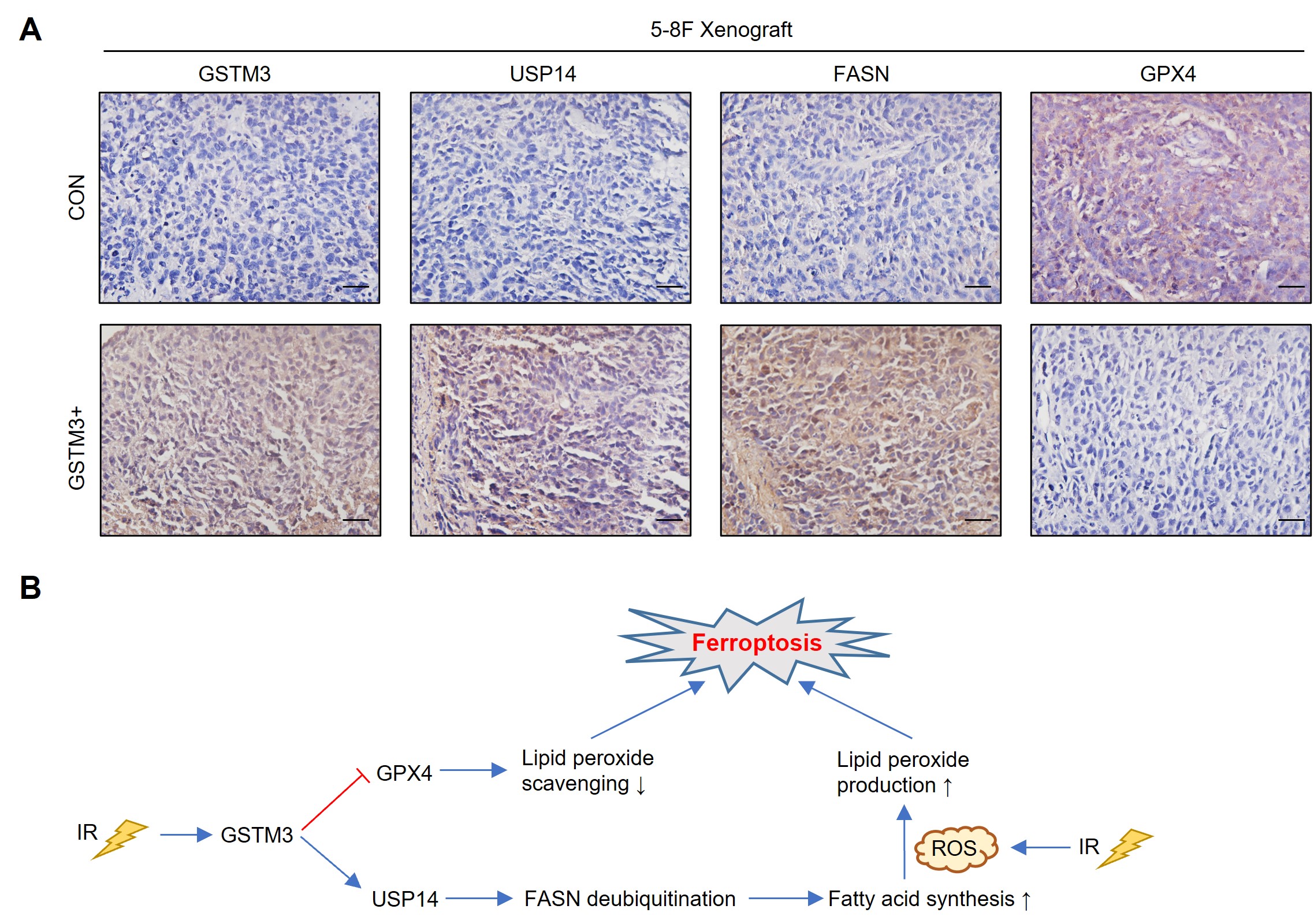
**

**Supplementary Fig 8.** **Proposed mechanism of GSTM3 improving IR-induced ferroptosis in NPC.** **A** IHC staining of GSTM3, USP14, FASN, and GPX4 in the subcutaneous tumor xenograft model. Scale bars: 50 μm. **B** The schematic diagram illustrates the proposed mechanisms by which GSTM3 improves IR-induced ferroptosis in NPC.

**
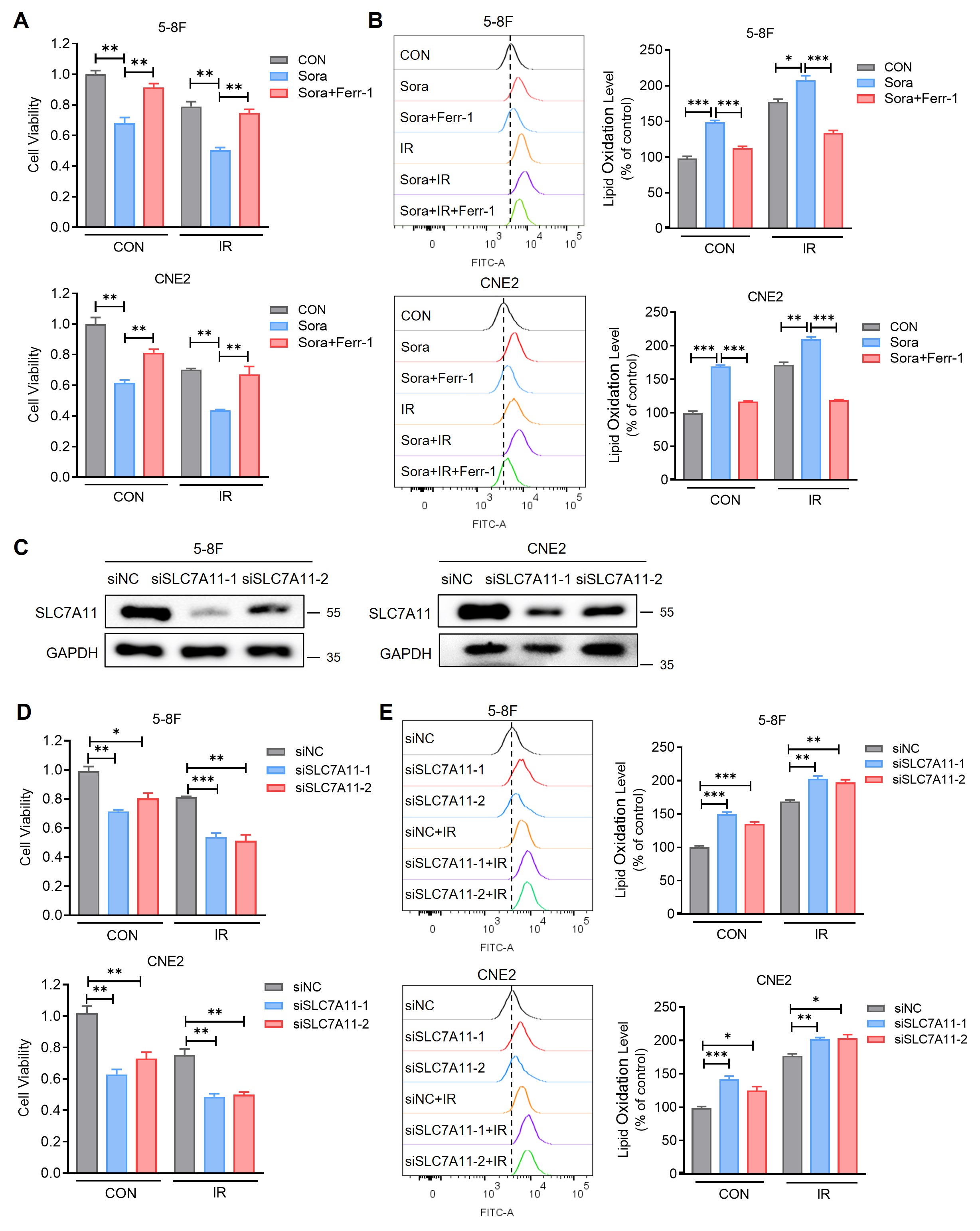
**

**Supplementary Fig 9.** **Sorafenib induces ferroptosis and enhances radiosensitivity by targeting SLC7A11 in NPC.** **A, B** The cell viability (A) and lipid peroxidation levels (B) in 5-8F and CNE2 cells after treatment with sorafenib for 24 h in the absence or presence of ferrostatin-1. **C** Western blot analysis of SLC7A11 protein in NPC cells transfected with siNC or siSLC7A11. **D, E** 5-8F and CNE2 cells transiently transfected with siNC or siSLC7A11 were treated with 6 Gy IR. After 24 h, the cell viability (D) in each group was determined using Cell Counting Kit-8, and the lipid peroxidation levels (E) were assessed via C11-BODIPY 581/591 staining. Data are presented as the mean ± SEM. Comparisons were made using the two-tailed Student’s *t*-test. **p* < 0.05; ***p* < 0.01; ****p* < 0.001.

**
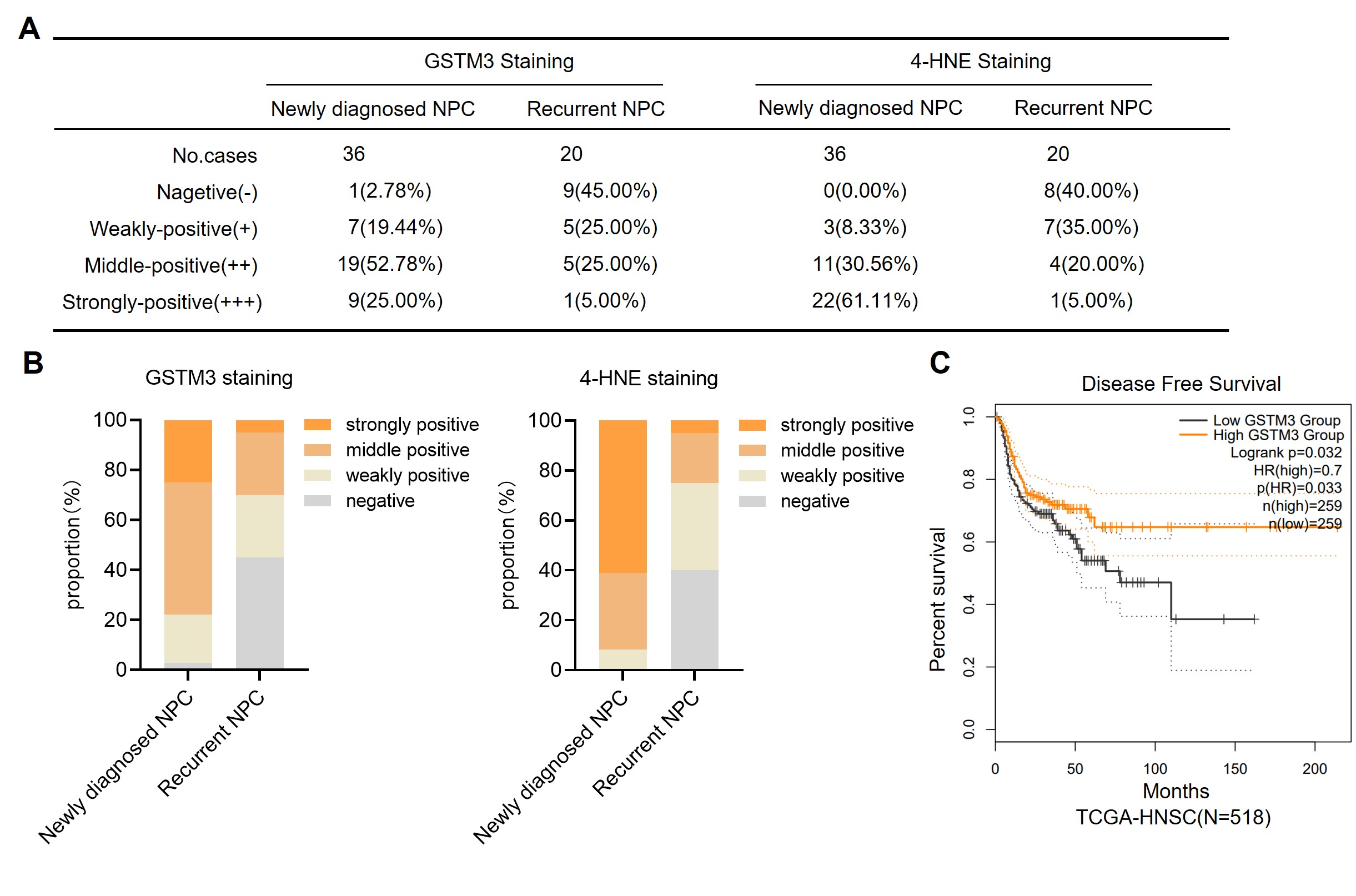
**

**Supplementary Fig 10.** **Low GSTM3 and 4-HNE abundance predicts tumor relapse and poor prognosis.** **A** The summary sheet of GSTM3 and 4-HNE staining intensity in patients with newly diagnosed NPC (n=36) or recurrent NPC (n=20). **B** Correlations of NPC recurrence status with the levels of GSTM3 or 4-HNE expression as detected by IHC. **C** Kaplan–Meier analysis of disease-free survival grouped by the GSTM3 expression of head and neck squamous cell carcinomas (HNSC) cohort based on TCGA database.

**Supplementary Table 1.** List of siRNA sequences.

| Gene |  | siRNA sequence (5’ to 3’) |  |  |
| --- | --- | --- | --- | --- |
| si-GSTM3#1 | Sense | CGUCUAUGGUUCUCGGGUATT |  |  |
|  | Antisence | UACCCGAGAACCAUAGACGTT |  |  |
| si-GSTM3#2 | Sense | CAACAUGUGUGGUGAGACUTT |  |  |
|  | Antisence | AGUCUCACCACACAUGUUGTT |  |  |
| si-USP14#1 | Sense | GCUUCAGCGCAGUAUAUUATT |  |  |
|  | Antisence | UAAUAUACUGCGCUGAAGCTT |  |  |
| si-USP14#2 | Sense | GCAUAUCGCUUACGUUCUATT |  |  |
|  | Antisence | UAGAACGUAAGCGAUAUGCTT |  |  |
| si-GPX4#1 | Sense | GACCGAAGUAAACUACACUTT |  |  |
|  | Antisence | AGUGUAGUUUACUUCGGUCTT |  |  |
| si-GPX4#2 | Sense | CAGGGAGUAACGAAGAGAUTT |  |  |
|  | Antisence | AUCUCUUCGUUACUCCCUGTT |  |  |
| si-FASN#1 | Sense | GCACCAAUACAGAUGGCUUTT |  |  |
|  | Antisence | AAGCCAUCUGUAUUGGUGCTT |  |  |
| si-FASN#2 | Sense | GGGACAGUGCAUCAAAGAATT |  |  |
|  | Antisence | UUCUUUGAUGCACUGUCCCTT |  |  |
| si-SLC7A11#1 | Sense | GCUGAAUUGGGAACAACUATT |  |  |
|  | Antisence | UAGUUGUUCCCAAUUCAGCTT |  |  |
| si-SLC7A11#2 | Sense | GCAGUUGCUGGGCUGAUUUTT |  |  |
|  | Antisence | AAAUCAGCCCAGCAACUGCTT |  |  |

**Supplementary Table 2.** List of PCR primers sequences.

| Gene |  | PCR primer sequence (5’ to 3’) |
| --- | --- | --- |
| PTGS2 | Forward | CTGGCGCTCAGCCATACAG |
|  | Reverse | CGCACTTATACTGGTCAAATCCC |
| GSTM3 | Forward | TACCTCTTATGAGGAGAAACGGT |
|  | Reverse | AGGAAAGTCCAGGTCTAGCTTG |
| USP14 | Forward | ATGCCGCTCTACTCCGTTACT |
|  | Reverse | GCCTTGAATACCATTGGAGGTTC |
| FASN | Forward | AAGGACCTGTCTAGGTTTGATGC |
|  | Reverse | TGGCTTCATAGGTGACTTCCA |
| GPX4 | Forward | GAGGCAAGACCGAAGTAAACTAC |
|  | Reverse | CCGAACTGGTTACACGGGAA |
| ANPEP | Forward | TTCAACATCACGCTTATCCACC |
|  | Reverse | AGTCGAACTCACTGACAATGAAG |
| CHAC1 | Forward | GGTGACGCTCCTTGAAGATCAT |
|  | Reverse | TCAGTGGTTGGTCAGGAGCAT |
| GGT7 | Forward | CCTTGTGTTTGGGTATCGTGG |
|  | Reverse | TTCGTCGGATGTCATGTACCA |
| GSTM2 | Forward | TGTGCGGGGAATCAGAAAAGG |
|  | Reverse | GCTGGGCTCAAATACTTGGTTT |
| ACSBG1 | Forward | ACACTGTGCATCGGATGTTCT |
|  | Reverse | AGGAGATGTGTTCCCACTTGT |
| ALOX15B | Forward | GACCCTGCTATACCAGAGCC |
|  | Reverse | ACCAGTCCCACTTGTCATCAG |
| APOE | Forward | GTTGCTGGTCACATTCCTGG |
|  | Reverse | GCAGGTAATCCCAAAAGCGAC |
| ELOVL3 | Forward | CTGTTCCAGCCCTATAACTTCG |
|  | Reverse | GAATGAGGTTGCCCAATACTCC |
| PLA2G3 | Forward | TATGGCATCCGAAACTACCGA |
|  | Reverse | GATGGAGTCGTGCTGATTCTG |
| PLA2G4A | Forward | AATACTGCACAATGCCCTTTACC |
|  | Reverse | GCTTCCAAATAAGTCGGGAGC |
| β-actin | Forward | TCAAGATCATTGCTCCTCCTGA |
|  | Reverse | CTCGTCATACTCCTGCTTGCTG |

**Supplementary Table 3.** List of antibodies used in this study.

| Antigens | Manufacturer | Application |
| --- | --- | --- |
| GSTM3 | Proteintech, 15214-1-AP | WB(1:2000); IHC(1:100) |
| USP14 | Proteintech, 14517-1-AP | WB(1:2000) |
| FASN | Proteintech, 10624-2-AP | WB(1:5000); IF(1:100); IHC(1:200) |
| GPX4 | Proteintech, 67763-1-Ig | WB(1:5000); IF(1:500); IHC(1:500) |
| SLC7A11 | Proteintech, 26864-1-AP | WB(1:1000) |
| ACSL4 | Proteintech, 66617-1-Ig | WB(1:5000) |
| TF | Proteintech, 17435-1-AP | WB(1:2000) |
| FTL | Proteintech, 10727-1-AP | WB(1:5000) |
| γ-H2AX | Proteintech, 10856-1-AP | WB(1:5000) |
| β-Tubulin | Proteintech, 10068-1-AP | WB(1:7500) |
| GSTM3 | Novus Biologicals, NBP3-04375 | IF(1:200) |
| USP14 | Proteintech, 67746-1-Ig | IF(1:200); IHC(1:100) |
| 4-HNE | Abcam, ab46545 | IHC(1:200) |
| PCNA | Proteintech, 10205-2-AP | IHC(1:500) |
| FLAG | Sigma-Aldrich, F1804 | IP(3μg) |
| HA | Dia-An Biotech, 2063 | IP(3μg) |

**Supplementary Table 4.** Radiobiological Parameters of NPC cells in different group.

| Cell line | Group | N | D0 | Dq | SF2 |
| --- | --- | --- | --- | --- | --- |
| 5-8F | CON | 2.472 | 1.511 | 1.368 | 0.535 |
|  | Ferr-1 | 3.115 | 2.430 | 2.761 | 0.835 |
| HONE1 | CON | 2.211 | 1.142 | 0.906 | 0.344 |
|  | Ferr-1 | 2.395 | 2.201 | 1.922 | 0.709 |
| CNE2 | CON | 2.735 | 0.978 | 0.984 | 0.315 |
|  | Ferr-1 | 2.872 | 1.818 | 1.918 | 0.687 |

N: extrapolation number; D0: mean lethal dose; Dq: quasi-threshold dose; SF2: surviving fraction at 2 Gy.

**Supplementary Table 5.** Association of the expression of GSTM3 with clinical features in 56 patients with NPC.

|  | GSTM3 expression | |  |
| --- | --- | --- | --- |
| Variables | Low (n=22) | High (n=34) | *p*-value |
| **Ages** |  |  |  |
| ≥50 | 10 (43.48%) | 13 (56.52%) | 0.5917 |
| ＜50 | 12 (36.36%) | 21 (63.64%) |  |
| **Gender** |  |  |  |
| Male | 18 (36.73%) | 31 (63.27%) | 0.3011 |
| Female | 4 (57.14%) | 3 (42.86%) |  |
| **Pathology** |  |  |  |
| Differentiated | 3 (28.57%) | 5 (71.43%) | 0.5349 |
| Undifferentiated | 19 (40.82%) | 29 (59.18%) |  |
| **Tumor Stage** |  |  |  |
| T1-T2 | 7 (43.75%) | 9 (56.25%) | 0.6653 |
| T3-T4 | 15 (37.50%) | 25 (62.50%) |  |
| **Node Stage** |  |  |  |
| N0-N1 | 5 (26.32%) | 14 (73.68%) | 0.1544 |
| N2-N3 | 17 (45.95%) | 20 (54.05%) |  |
| **Clinical stages** |  |  |  |
| I-II | 3 (23.08%) | 10 (76.92%) | 0.1721 |
| III-IV | 19 (44.19%) | 24 (55.81%) |  |
| **Metastasis** |  |  |  |
| Yes | 9 (64.29%) | 5 (64.29%) | **0.0270** |
| No | 13 (30.95%) | 29 (69.05%) |  |
| **Recurrence** |  |  |  |
| Yes | 14 (70.00%) | 6 (30.00%) | **0.0005** |
| No | 8 (22.22%) | 28 (77.78%) |  |

The *P-*values were determined using the two-tailed χ^2^ test.

**Supplementary Table 6.** Association of the expression of 4-HNE with clinical features in 56 patients with NPC.

|  | 4-HNE expression | |  |
| --- | --- | --- | --- |
| Variables | Low (n=18) | High (n=38) | *p*-value |
| **Ages** |  |  |  |
| ≥50 | 8 (34.78%) | 15 (65.22%) | 0.7240 |
| ＜50 | 10 (30.30%) | 23 (69.70%) |  |
| **Gender** |  |  |  |
| Male | 16 (32.65%) | 33 (67.35%) | 0.8288 |
| Female | 2 (28.57%) | 5 (71.43%) |  |
| **Pathology** |  |  |  |
| Differentiated | 4 (50.00%) | 4 (50.00%) | 0.2428 |
| Undifferentiated | 14 (29.17%) | 34 (70.83%) |  |
| **Tumor Stage** |  |  |  |
| T1-T2 | 6 (37.50%) | 10 (62.50%) | 0.5872 |
| T3-T4 | 12 (30.00%) | 28 (70.00%) |  |
| **Node Stage** |  |  |  |
| N0-N1 | 9 (47.37%) | 10 (52.63%) | 0.0804 |
| N2-N3 | 9 (24.32%) | 28 (75.68%) |  |
| **Clinical stages** |  |  |  |
| I-II | 3 (23.08%) | 10 (76.92%) | 0.4244 |
| III-IV | 15 (34.88%) | 28 (65.12%) |  |
| **Metastasis** |  |  |  |
| Yes | 6 (42.86%) | 8 (57.14%) | 0.3216 |
| No | 12 (28.57%) | 30 (71.43%) |  |
| **Recurrence** |  |  |  |
| Yes | 15 (75.00%) | 5 (25.00%) | **<0.0001** |
| No | 3 (8.33%) | 33 (91.67%) |  |

The *P-*values were determined using the two-tailed χ^2^ test.
